# Comparison of secretory signal peptides for heterologous protein expression in microalgae: Expanding the secretion portfolio for *Chlamydomonas reinhardtii*

**DOI:** 10.1371/journal.pone.0192433

**Published:** 2018-02-06

**Authors:** João Vitor Dutra Molino, João Carlos Monteiro de Carvalho, Stephen Patrick Mayfield

**Affiliations:** 1 Department of Biochemical and Pharmaceutical Technology, School of Pharmaceutical Sciences, University of São Paulo, São Paulo, São Paulo, Brazil; 2 California Center for Algae Biotechnology, Division of Biological Sciences, University of California, San Diego, California, United States of America; Youngstown State University, UNITED STATES

## Abstract

Efficient protein secretion is a desirable trait for any recombinant protein expression system, together with simple, low-cost, and defined media, such as the typical media used for photosynthetic cultures of microalgae. However, low titers of secreted heterologous proteins are usually obtained, even with the most extensively studied microalga *Chlamydomonas reinhardtii*, preventing their industrial application. In this study, we aimed to expand and evaluate secretory signal peptides (SP) for heterologous protein secretion in *C*. *reinhardtii* by comparing previously described SP with untested sequences. We compared the SPs from arylsulfatase 1 and carbonic anhydrase 1, with those of untried SPs from binding protein 1, an ice-binding protein, and six sequences identified *in silico*. We identified over 2000 unique SPs using the SignalP 4.0 software. mCherry fluorescence was used to compare the protein secretion of up to 96 colonies for each construct, non-secretion construct, and parental wild-type cc1690 cells. Supernatant fluorescence varied according to the SP used, with a 10-fold difference observed between the highest and lowest secretors. Moreover, two SPs identified *in silico* secreted the highest amount of mCherry. Our results demonstrate that the SP should be carefully selected and that efficient sequences can be coded in the *C*. *reinhardtii* genome. The SPs described here expand the portfolio available for research on heterologous protein secretion and for biomanufacturing applications.

## Introduction

Microscopic eukaryotic green algae are suitable candidates for the production of several bio-derived products, such as, pigments, proteins, lipids, carbohydrates, vitamins, and anti-oxidants [1]. Microalgae are an appealing biomanufacturing platform, because they are solar-based fast-growing organisms with low nutrient requirements [2]. These attributes led to a global market value of €2.4 bn in 2011, which is low with respect to food commodities, but represents the result of steady growth over the last two decades [3]. Although the microalgae market is mostly composed of non-recombinant strains, the development of recombinant products, such as protein vaccines, therapeutic antibodies, and industrial enzymes, can add value to the global market. Protein-based products can exploit the low-cost advantage of microalgae systems to produce cheap recombinant proteins, such as industrial enzymes [4]. Even the production of protein-based therapeutics, for which cultivation cost might be a less significant factor [5], microalgae can be used to produce unique therapeutic proteins, such as immunotoxin [6]. The unicellular microalga *Chlamydomonas reinhardtii* has been shown to produce distinct proteins that may be used for specific purposes, such as therapeutic, nutraceuticals, and industrial enzymes [7,8]. As plants, microalgae also have another interesting feature; the availability of three editable genomes [6,9–11]. Among the editable genomes, the chloroplast and nuclear genomes have potential biotechnological applications. Nevertheless, despite the ability of chloroplasts to produce active complex proteins such as antibody-drug conjugates [6], nuclear protein expression permits the use of interesting features. Nuclear expression enables proteins to be targeted to organelles, or secreted into media, and to undergo post-translational modification, such as glycosylation [9,12]. Furthermore, important nuclear modification technologies are being developed, expanding the molecular toolkit for nucleus transformations, potentially impacting the yield of nuclear proteins, and thus, the commercial exploitation of this system in biotechnology [10,13,14].

An interesting opportunity for recombinant protein production in microalgae is through the secretion of proteins into the extracellular matrix [15–17]. Secretion allows the use of standard bioprocess steps, such as continuous centrifugation [18], to separate the compound-complex cells from the extracellular compound-simple matrix, impacting downstream cost [19]. Besides the downstream advantages, secretion enables the use of perfusion technologies, potentially impacting the daily productivity and the facility footprint [20], increasing the productive life cycle of the cell and enabling high-density cultures. In addition, the typical low cost of media associated with microalgae cultivation also contributes to perfusion technology, by reducing the constraints related to the high volume of media used in perfusion, which can represent 12% of the production cost [20]. In addition to the advantages of the production process, glycosylation modification in the secretory pathway might play an important role in protein function and stability [21]. Despite their inherent advantages, secretion studies in microalgae expression systems are scarce and must be pursued. Nevertheless, in an important recent breakthrough, Ramos-Martinez et al. (2017) fused synthetic glycomodules to a fluorescent protein, subsequently increasing the secreted protein yield 12-fold to a final concentration of 15 mg L^-1^, demonstrating the potential to obtain higher secretion efficiencies in microalgae [21].

Recombinant proteins are secreted by the presence of SPs to the N-terminal portion of the protein [22]. In fact, heterologous protein secretion strategies use different SPs, which are validated in the expression system used, and originate from either the host-secreted protein repertoire [8,21,23–25] or different species [25,26]. Interestingly, Knappskog et al. (2007) showed that the use of alternative SPs affects the efficiency of protein secretion [27]; therefore, SP selection affects the overall yield of secreted proteins in an expression system. SPs to secret recombinant protein are usually attained from naturally secreted proteins, on transient proteins of the endoplasmic reticulum, Golgi apparatus, and cytoplasmic membrane proteins [28]. Even SPs from proteins residents of the endoplasmic reticulum are suitable, such as BiP1, if the retention signals are not retained on the final sequence. In a study directly comparing the efficiency of SPs, Kober et al. (2013) compared 17 SPs in CHO cells, which were selected based on the results of a literature search for promising SPs. Each SP promoted a different protein secretion efficiency, with a wide variation observed among peptides, reaching a difference of more than 100-fold between the lowest and highest secretors [28]. Despite the importance of SPs, studies in the literature comparing the efficiency of SPs are lacking [28], with the literature on *C*. *reinhardtii* being no exception. Out of the nine studies published on recombinant protein secretion in *C*. *reinhardtii*, only that of Lauersen et al. (2013) compared the efficiency of two SPs [8,9,21,25,29–33], and reported a difference of 84% on the amount of secreted protein between the SPs tested. However, *C*. *reinhardtii* secretes a variety of proteins, and the ability of only a few SPs to promote heterologous protein secretion has been evaluated [34].

To advance protein secretion technology in *C*. *reinhardtii* expression systems, we compared the fusion of 10 SPs to the mCherry fluorescent protein. We aimed to directly compare SPs for use in the *C*. *reinhardtii* system, to increase options and to support SP selection for protein secretion in this microalga. Therefore, we selected four SPs described in the scientific literature [8,25,35,36] and six from over 2000 unique *in silico* SPs identified by SignalP 4.0 software [37]. SignalP 4.0 predicted possible SPs from the *C*. *reinhardtii* protein sequence dataset obtained from the DOE Joint Genome Institute [38]. In this study, we identified five new, previously undescribed, functional SPs, and to our knowledge, this represents the first description of an ice-binding protein SP in heterologous protein secretion. Furthermore, two of the newly identified SPs outperformed the evaluated sequences, demonstrating the potential contained within the unevaluated part of the *C*. *reinhardtii* genome. Efficient recombinant protein secretion represents a milestone towards the industrial application of transgenic microalgae, and the results presented herein are a step in that direction.

## Results

### Secretion efficiency of SPs

We evaluated 10 SPs based on their secretion efficiency (Table 1). We selected one SP from each of four types of secreted protein identified in the literature: binding protein 1 –BiP1 [35]; arylsulfatase 1 –ARS1 [8]; carbonic anhydrase 1 –CAH1 [25], and ice-binding protein 1 –IBP1 [36]. Each selected SP has the capacity to direct protein synthesis to the secretory pathway of its natural protein, and ARS1 and CAH1 were previously demonstrated to secrete heterologous proteins [8,25]. IBP1 originates from an ice-binding protein from a *Chlamydomonas sp*. identified in the Artic [36], and BiP1 is an endoplasmic reticulum protein from *C*. *reinhardtii* [35].

**Table 1:** Characteristics of the signal peptide tested.

Construct	Signal peptide sequence	Available protein information					
		Identification/Blast similarity information	Protein size (aa)	E-value	Ident. (%)	Cover (%)	Source
**pJP26**	MAQWKAAVLLLALACASY	Binding protein 1 (BiP1)	656				[35]
**pJP22**	MHARKMGALAVLAVACLAAVASVAHA	Arylsulfatase 1 (ARS1)	654				[8]
**pJP28**	MARTGALLLVALALAGCAQA	Carbonic anhydrase (CAH1)	377				[25]
**pJP29**	MPSSSMKLFAALLIACMAQTSMA	Ice-binding protein 1 (IBP1)	353				[36]
**pJP30**^a^	MRRAIALGVGLALLGLLLPGSLA	Glycoside-hydrolase-like protein, 1,3-α-glucosidase [*Momordica charantia*] catenatum]	1641	0.0	50	54	This work
**pJP31**^a^	MPSTPVAAALRALLCASLLGSLHIARA	Expressed protein [*Chlorella variabilis*], Sulfotransferase family	361	4e-16	27	69	This work
**pJP32**^a^	MTLRLAQLALATLGVLLLVLAPMPALS	Possible cell wall protein, SAD1p [*Chlamydomonas reinhardtii*]	4531		80		This work
**pJP33**^a^	MARRLLLALALAAVLGLAHA	Prolyl 4-hydroxylase [*Volvox carteri f*. *nagariensis*]	273	4e-77	64	74	This work
**pJP34**^a^	MMSSLISSRVAALLPALQHASG	Succinate dehydrogenase assembly Fact. 4, mitochondrial-like [*Glycine max*]	151	4e-10	61	34	This work
**pJP35**^a^	MRGIIAAYTSATLLALLLVTWLTHSSA	Mitogen activated protein kinase kinase kinase 7 [*C*. *reinhardtii*]	1339	0.0	100	100	This work

Ident, Identity; aa, amino acid length

^**a**^ The respective proteins of the *in silico* identified signal peptides were blast at NCBI and Uniprot database, and the information used to infer similarity.

SignalP4.0 is used to predict the SPs in protein sequence datasets [37]. The *C*. *reinhardtii* protein repository from the DOE Joint Genome Institute Databank (Assembly v4.0, Dataset—Chlre4_best_proteins.fasta) was submitted for SignalP4.0 analysis, generating 8556 possible SPs, with 2221 unique sequences (S1 Dataset). We selected six SPs identified *in silico* from the phylogenetic tree (S1 Fig) generated with the SP dataset, thus completing the set of SPs evaluated. We selected SPs from different branches on the phylogenetic tree. A protein BLAST search of the selected proteins confirmed the choice of non-described SPs.

The constructs containing SPs were termed pJP and consisted of an expression cassette derived from pBR9 [8] using a single protein expression strategy, fusing the antibiotic selection marker (bleomycin) with the gene of interest by a self-cleavage peptide (FMDV-2A [S5 Fig]). The following modifications were included in the pBR9 cassette to establish high titers: the chimeric promoter HSP70A-RBSC2; RBSC2 introns 1, 2, and 3; and the RBSC2 3’ untranslated region (UTR) as a transcriptional terminator [29]. The modified pBR9 was termed pAH04, and was used as a non-secreting control (details in Materials and Methods). All 10 pJP construct sequences were derived from pAH04 by the insertion of a SP at the 5’ position of the gene of interest by SLICE reactions; the sequence was subsequently verified. A cell walled strain, cc1690, was transformed with the expression vectors, and screened for the presence of mCherry fluorescence.

**Fig 1:**
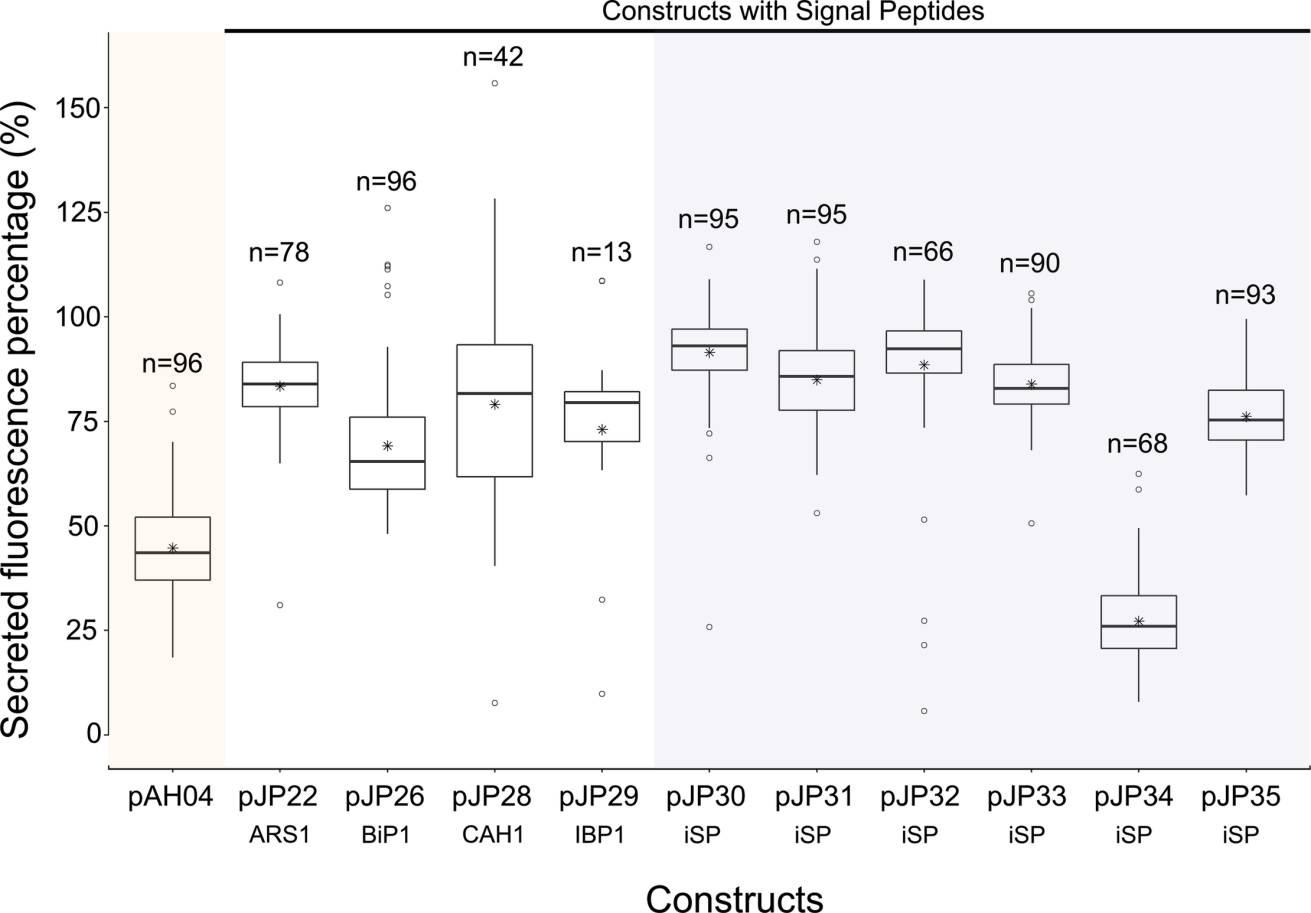
Comparison of relative mCherry fluorescence in the supernatant of the studied expression vector constructs. mCherry fluorescence was measured in the whole culture sample, compared with mCherry fluorescence in the supernatant, and is expressed as a percentage. mCherry fluorescence was measured from 96 individual colonies grown in a deep-well plate in TAP media for 7 days under constant illumination and agitation. Data presented in the boxplot were collected from colonies where the total fluorescence signal was higher than that of the auto fluorescence of the parental wild-type cc1690, within three standard deviations. pAH04 –construct without SP; pJP22 –construct with arylsulfatase 1 SP; pJP26 –construct with binding protein 1 SP; pJP28 –construct with carbonic anhydrase 1 SP; pJP29 –construct with ice-binding protein 1 SP; pJP30-35 –construct with *in silico* identified SP (iSP). n–number of positive signals obtained for each construct. * represents the average result. ○ represents the outliers.

To determine the frequency of true positives and the secretion capacity of each expression vector, 96 colonies of each construct were cultivated mixotrophically in a tris-acetate phosphate (TAP) medium. The fluorescence of the whole culture, and that of the supernatant, was read for all 96 cells. The parental cc1690 strain was used as a blank, and secretions from pAH04 cells were used as a negative control. We checked all cell growths by chlorophyll fluorescence, and a low relative standard deviation was observed (<10%). The fluorescence results are accessible in the S2 Dataset. mCherry secretion by each construct was assessed by the proportion of mCherry fluorescence in the supernatant relative to that in the total culture (Fig 1).

A one-way ANOVA was conducted to compare the relative secretory capacity of each construct for the non-secreting pAH04 construct and the theoretical secreting pJP constructs. Nine constructs had a significant effect on mCherry secretion (p < 0.05) [F(10, 821) = 172.5, p = <2e-16]. Post-hoc comparisons using Tukey`s HSD test indicated that the mean score for the non-secreting construct pAH04 (M = 44.7, SD = 11.5) was significantly different from that of the secreting pJP constructs. Despite a significant difference observed between the pJP34 (M = 27.2, SD = 11.0) and pAH04 constructs, the mean value of the former was lower than that of pAH04 indicating non-secretion by this vector. Taken together, these results suggest that pJP constructs promote secretion. Specifically, our results suggest that SignalP4.0 could identify true SPs from a *C*. *reinhardtii* protein sequence dataset, although this was not achieved with 100% accuracy, as expected [37].

**Fig 2:**
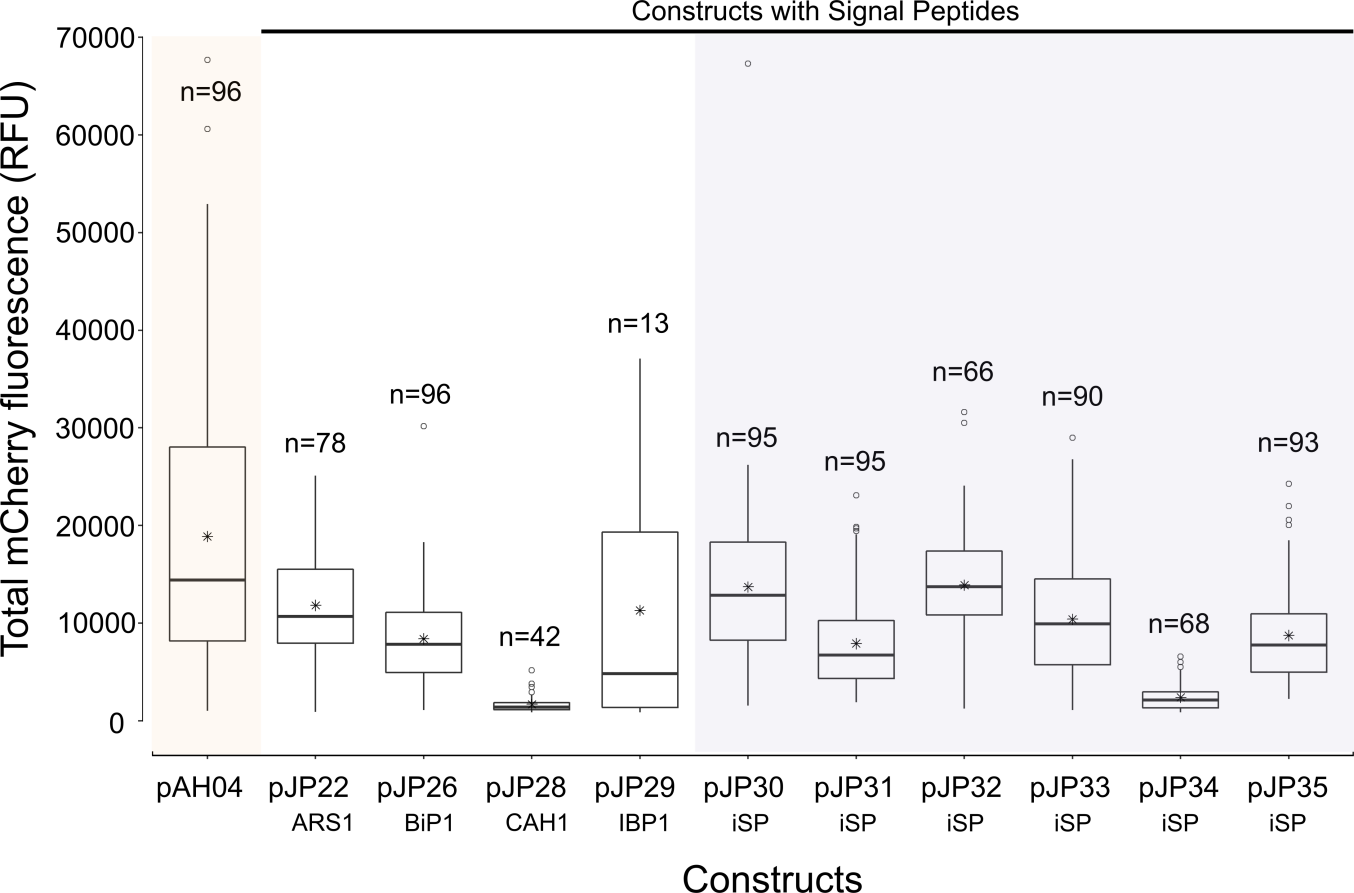
Comparison of mCherry fluorescence among expression vector constructs in the total culture. mCherry fluorescence was measured in the total culture sample. mCherry fluorescence was measured from 96 individual colonies, grown in a deep-well plate in TAP media for 7 d under constant illumination and agitation. Data presented in the boxplot were collected from colonies in which the total fluorescence signal was higher than that of the auto fluorescence signal of the parental wild-type cc1690, above three standard deviations. pAH04 –construct without SP; pJP22 –construct with arylsulfatase 1 SP; pJP26 –construct with binding protein 1 SP; pJP28 –construct with carbonic anhydrase 1 SP; pJP29 –construct with ice-binding protein 1 SP; pJP30-35 –construct with *in silico* identified SP (iSP). n–number of positive results obtained for each construct. * represents the average result. ○ represents the outliers. No normalization was conducted for mCherry fluorescence.

Although nine pJP constructs could promote secretion, the mCherry fluorescence signal varied significantly [F(10, 821) = 34.36, p = <2e-16] among constructs. Tukey`s HSD test indicated that the mean score for the non-secreting construct pAH04 (M = 18,881.4, SD = 14,418.2) was significantly higher than that of the highest secreting construct pJP32 (M = 13,908.1, SD = 5933.4). Furthermore, there was wide variation in the mCherry fluorescence detected among secreting pJP transformants (8-fold increase from low [pJP28] to high [pJP32] secretors), as shown in Fig 2. In general, the non-secreting transformant outperformed secreting transformants in terms of mCherry production, nevertheless, considering downstream purification advantages of secreted proteins, this represents a low margin (<30% for pJP30 and pJP32). Still, pJP28 (construct with CAH1 SP) and pJP34 (construct with non-functional SP) generated cells with low mCherry expressing ability, indicating that SP interfered with mCherry production in our transformants. We did not normalize the mCherry fluorescence data, to allow any effect on cell growth to be computed together in our analysis, since the aim was to detect the construct that led to a higher overall production.

**Fig 3:**
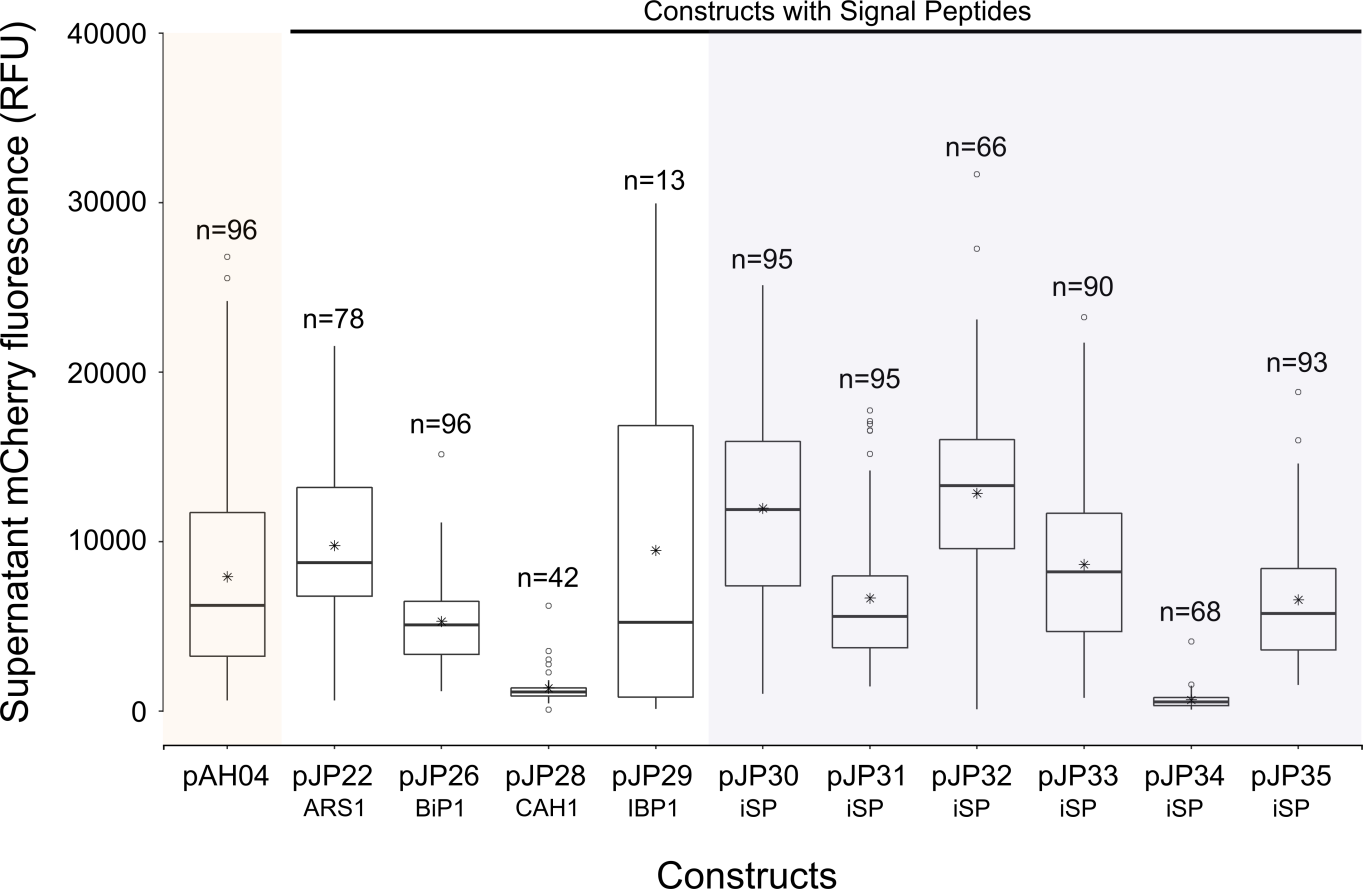
Comparison of mCherry fluorescence in the supernatant of different constructs. mCherry fluorescence was measured in supernatant. mCherry fluorescence was measured from 96 individual colonies, grown in a deep-well plate in TAP media for 7 d under constant illumination and agitation. Data presented in the boxplot were collected from colonies where the total fluorescence signal was higher than the auto-fluorescence of the parental wild-type cc1690, above three standard deviations. pAH04 –construct without SP; pJP22 –construct with arylsulfatase 1 SP; pJP26 –construct with binding protein 1 SP; pJP28 –construct with carbonic anhydrase 1 SP; pJP29 –construct with ice-binding protein 1 SP; pJP30-35 –construct with *in silico* identified SP (iSP). n–number of positive results obtained for each construct. * represents the average result. ○ represents the outliers. No normalization was conducted for mCherry fluorescence.To confirm this hypothesis, a positive colony of each construct with the highest fluorescence was cultivated in 50 mL of TAP media for 7 d, and mCherry fluorescence was determined in both the total cultures and in the supernatant (S2 Fig). The supernatant percentage of mCherry fluorescence in the pAH04 strain cultivated in the flask was lower relative to that of the pAH0A strain cultivated on the plate (from 42% on the plate to ~8.5% in the flask), which was consistent with the culture condition hypothesis. Although the test in the flask presented a lower noise, it lacks the throughput to test several colonies, an important feature when comparing different construct designs. Since transformation is based mainly on a random insertion by non-homologous end joining (NHEJ) [39], colonies presented a wide range of expression efficiency, from a relative standard deviation of 42.7% to 102.7% (S1 Table). Therefore, we chose the 96 well plate assay to compare constructs efficiency since it could prevent sampling bias.

Direct comparison of secretion among the 11 constructs revealed significant effects, as determined by ANOVA, at the p < 0.05 level [F(10, 821) = 43.5, p = <2e-16], and the mCherry fluorescence results can be observed in Fig 3. Post-hoc comparison using Tukey HSD test indicated that mCherry fluorescence in the supernatant of the non-secreting pAH04 construct was significantly higher than that of three pJP constructs (pJP26, pJP28, and pJP34), and was not significantly different from that of two pJP constructs (pJP31 and pJP35). However, five pJP constructs exhibited significantly higher fluorescence than was observed in the pAH04 construct supernatant. The presumed secretion of mCherry by pAH04 strains might be explained by the sub-optimal cultivation protocol used. Sub-optimal cultivation, which may lead to an increased rate of cell lysis, together with the overall higher expression of pAH04 constructs, might explain this result.

**Fig 4:**
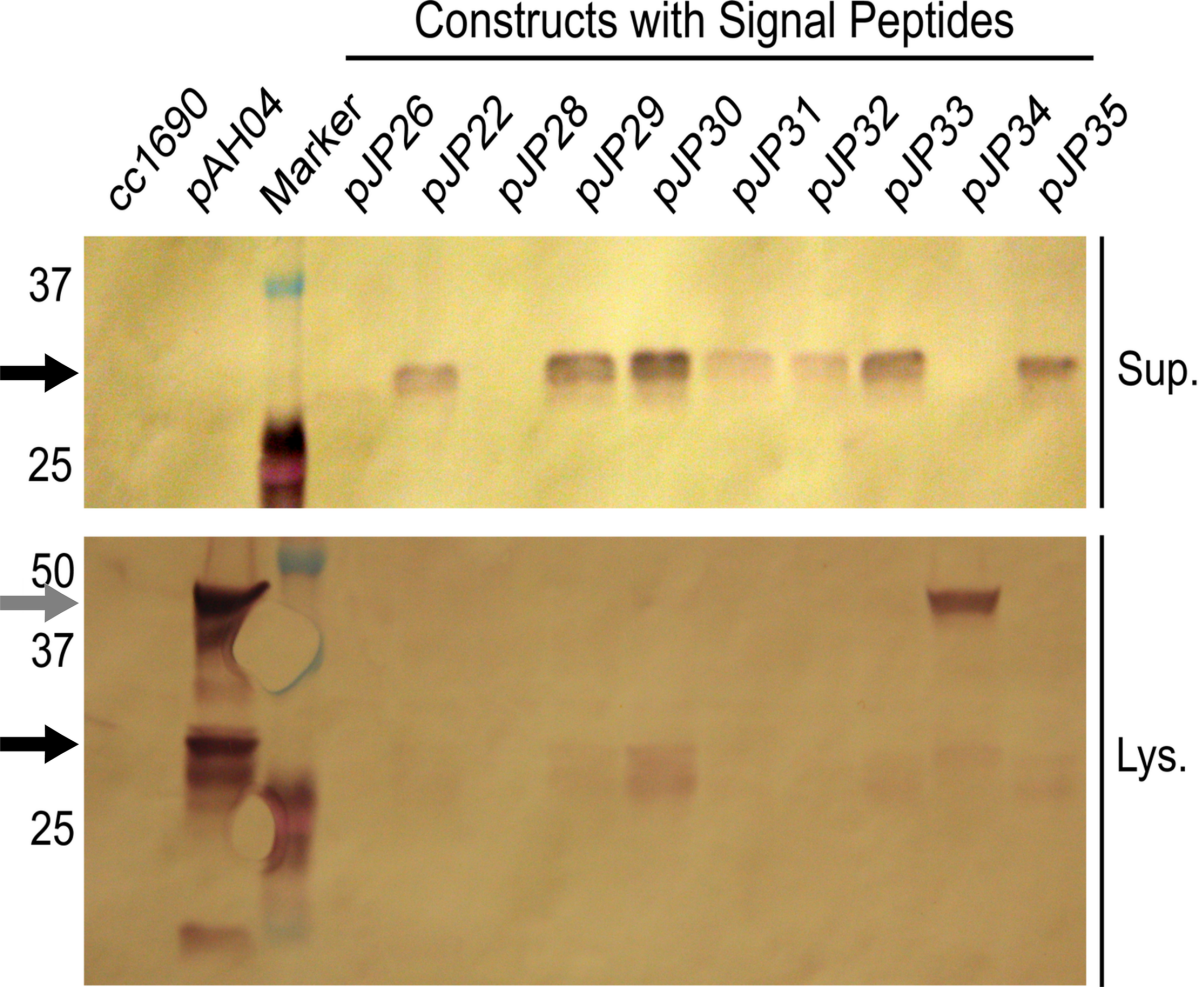
mCherry compartmentalization in immunoblotting analysis. Black arrow–mCherry bands, 27 KDa for unmodified and 29 KDa for post-translational modified; Grey arrow–mCherry band still fused with sh-ble and 2A autocleavage peptide, 46 KDa; Sup.–supernatant; Lys.–lysate, cc1690 –parental wild-type strain; pAH04 –construct without SP; pJP22 –construct with arylsulfatase 1 SP; pJP26 –construct with binding protein 1 SP; pJP28 –construct with carbonic anhydrase 1 SP; pJP29 –construct with ice-binding protein 1 SP; pJP30-35 –construct with *in silico* identified SP.

Western blotting was performed to confirm the presence of mCherry in the supernatant of pJP transformants and its absence in the supernatant of pAH04 (Fig 4). A single top producer for each strain was grown mixotrophically in a TAP medium for 7 days, and cell-free media and lysate were analyzed by western blotting. The parental strain cc1690 was used as a negative control. As expected, in the pAH04 strain, mCherry was found exclusively in the lysate; however, bands at different sizes were observed. Size differences among bands can be explained by incomplete separation of the fusion protein [8], different phosphorylation patterns [21], and protein degradation. The western blot band intensity directly correlated with the mCherry fluorescence, and was absent for low fluorescence readings of band intensity on western blot (S3 Dataset)

In accordance with the observed mCherry fluorescence, supernatant from the strains with higher secretion capacity generated visible bands on western blot analysis. The absence of bands for pJP26 (a construct with SP from BiP1) and pJP28 (a construct with SP from CAH1) can be explained by the low level of mCherry production from these strains (S2 Fig), at levels below the detection limit of western blot. The western blot represents just one data point, and the mCherry band absence is not absolute, since in 96 colonies for pJP26 and in 42 for pJP28 mCherry was detected in the supernatant by fluorescence reading (Fig 1). Conversely, as expected by analysis of relative fluorescence (Fig 1), pJP34 (a construct with a theoretical SP) lacked a band corresponding to mCherry in the supernatant; interestingly, a single band at 46 KDa was observed from the lysate, indicating conservation of the fused protein structure. A dim shadow could be observed in the lane corresponding to the pJP34 lysate at the correct mCherry size, implying that some cleavage occurs within the cell.

To complement secreted protein analysis, we performed a Coomassie stained polyacrylamide gel electrophoresis (PAGE) with sodium dodecyl sulfate (SDS), to assess protein complexity in the supernatant and to estimate the relative abundance of mCherry (S3 Fig). The protein context was observed in a lane-based manner, and the relative abundance was compared with those of other naturally secreted proteins. We concentrated the >10 KDa proteins present in supernatant samples from all transformants, and that of cc1690, by approximately 30-fold. Interestingly, the mCherry bands observable for the concentrated supernatant of pJP30 represented one of the most intense bands in the lane, computing to approximately 8% of the supernatant proteins. An increased percentage of proteins of interest in the media may help to reduce the costs associated with downstream steps. S3 Fig shows the intracellular protein complexity within the cells, which prevents the identification of mCherry bands, even from the pAH04 construct, which presented the strongest mCherry fluorescence signal and band on western blot.

**Fig 5:**
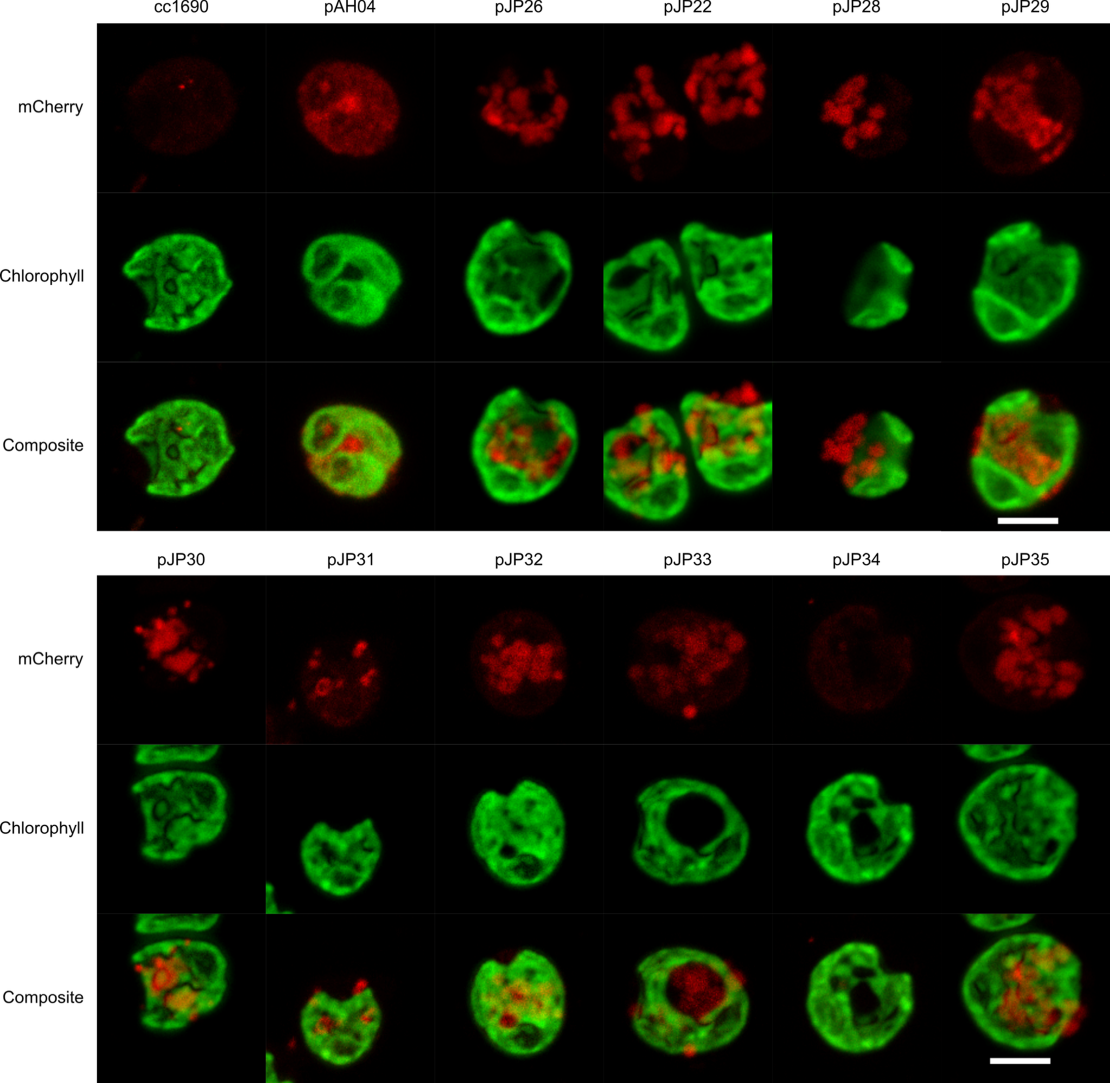
Live-cell fluorescence microscopy of the mCherry-expressing strains. The mCherry signal from the non-secreting pAH04 construct is distributed in the cytosol, while the secreting pJP transformants present a mCherry signal in vesicles. Live cells were plated on agar pads and images were acquired 0.4 -μm apart in each channel in the z-axis. Then, images were stacked using the Fiji software Z projects function, generating the final images. An argon laser at 543 nm was used to excite mCherry, and a spectral detector set at approximately 610–650 nm was used to detect emitted fluorescence. For chlorophyll, we used a laser at 405 nm for excitation, and a spectral detector set at 680 nm. cc1690 –parental wild-type strain; pAH04 –construct without SP; pJP22 –construct with arylsulfatase 1 SP; pJP26 –construct with binding protein 1 SP; pJP28 –construct with carbonic anhydrase 1 SP; pJP29 –construct with ice-binding protein 1 SP; pJP30-35 –construct with *in silico* identified SP. All images were processed identically. Scale bar = 5 μm.

Live-cell imaging was performed by confocal laser scanning microscopy to visualize the localization of mCherry proteins in the transformants (Fig 5). The overall fluorescence signals are similar in the images displayed (Fig 5). Nonetheless, the pattern displayed is different. The pAH04 strain exhibited a red fluorescence signal in the cytosol, representing active mCherry protein, distributed inside the cell, and limited by the cytoplasmic membrane, as reported previously [21,40,41]. Unlike pAH04 transformants, pJP strains exhibited condensed fluorescence signals in the cytoplasm, which is consistent with proteins trafficking inside the secretory pathway [9]. S4 Fig compares the different distribution pattern inside the cell. Nevertheless, fluorescence microscopy of the pJP34 strain presented a faded fluorescence signal, which was hardly distinguishable from that of the control cc1690 strain. A low level of active protein expression, combined with a protein diffused in the cytoplasm, may explain this result.

Taken together, these results indicate that with the expression vector design used, nine of the 10 SPs were functional and can be used to promote heterologous protein secretion.

## Discussion

The secretion of recombinant proteins into the extracellular environment by microalgae has been described in the literature [8,9,21,25,29–32,42,43], and indeed, research on efficient secretion strategies is important for the development of a recombinant protein expression system. Secretion allows the use of culture manufacturing strategies, such as perfusion, which have the potential for greater productivity and the use of smaller production facilities [20]. In fact, the use of perfusion in the production of monoclonal antibodies is estimated to reduce the overall production cost by 20% [20]. The elevated expense due to the requirement for high volumes of media in perfusion is attenuated in *C*. *reinhardtii*, which grow in a low-cost, defined media [44]. Nevertheless, secretion studies in microalgae have focused on individual SPs, or more recently, the use of glycomodules to enhance secretion efficiency [21]. Several other strategies are due to be explored [45–50], and different SPs will be evaluated [28]. In this study, we have shown that the efficiency of heterologous protein secretion from *C*. *reinhardtii* is deeply influenced by the SP used; our results showed that secretion varied 10-fold. Therefore, the construction of expression vectors should consider the choice of SP. We also aimed to address the low number of tested SPs described in the literature, and to directly compare their efficiency. Our results suggest that more efficient SPs may be encoded by the *C*. *reinhardtii* genome, as comparison of secretion efficiencies revealed that the two top secreting SPs, from constructs pJP30 and pJP32, were identified by SignalP 4.0 software using a genome-predicted protein dataset [38]. The SP from pJP30 came from a protein with similarity to glycoside-hydrolase-like proteins, more specifically with glucan 1,3-α-glucosidase (Table 1). The SP from pJP32 seems to be from a cell wall protein, due to its size and similarity to SAD1p, a structural constituent of the cell wall in *C*. *reinhardtii*.

It is important to note that in our study, two previously described efficient SPs were included as secretion-efficient references [8,25], to allow indirect comparisons to be made between our results and previously published data. Nevertheless, to allow comparisons among the SPs studied, the following variables were fixed: cell strain, expression vector design, and heterologous proteins secreted. Therefore, the parameters used in our experiment might not directly correlate with other experimental settings, since the cell strain might influence protein expression [51]. In addition, the SP context can influence its function [47] and the secretion efficiency varies in accordance with the expressed protein [46]. Nonetheless, the SPs were fused using an expression vector with a promising design, incorporating strategies described in the literature, such as codon-optimized synthetic genes [52], promoter fusion [53], and the use of native introns [29], to allow high levels of heterologous protein expression. We also selected a cell-walled wild-type strain that has been used since the mid-1950s, since most of the protein sequences in the dataset used in this study originated from this strain [38,54]. Nevertheless, the use of other *C*. *reinhardtii* strains will present different expression efficiencies [55], and they should be tested with the SPs used herein, since greater secretion efficiency might be obtained. Therefore, the SPs tested and presented herein will support other studies aiming to increase protein secretion, serving as a guide and portfolio of tested SPs.

The development of SPs for heterologous protein expression involves the identification of secreted proteins containing SPs [33,56] or an *in silico* approach [37]. From a methodological perspective, *in silico* analysis can generate a larger set of candidates, with a considerable certainty of functionality [37], and greater diversity, since it is independent of protein detection. Although more SPs are identified, not all are functional. In the present study, we identified more than 2000 unique SPs and tested six, of which five were functional, which is consistent with Matthew’s correlation coefficient of 0.9 in SignalP 4.0 [37]. Despite pJP34 SP being possibly nonfunctional, the results were inconclusive. Using a different expression vector design, in the context of a different amino acid sequence, pJP34 SP may exhibit functionality. Based on our results, we can conclude that pJP34 SP interferies with the 2A self-cleaving peptide, since western blot using sample lysates (Fig 4) revealed a single band at 46 KDa, a pattern that is consistent with the fused protein. Since the fused protein domains remain attached, the interaction with the signal recognition particle (SRP) could be hindered, and subsequent translational translocation to the endoplasmic reticulum blocked, since it is mediated by SRP [15]. Therefore, five of the six predicted SPs secreted heterologous proteins as determined by the results of the mCherry fluorescence (Fig 1) and western blot analyses (Fig 4), and one generated inconclusive results, due to our expression vector design. Another SP that might have been interfered with by our vector design is CAH1 SP. The low values for CAH1 SP are unexpected, since it was successfully used to secrete luciferase at high values (10 mg L^-1^) [25]. The reason for these low values is not clear, but there are major differences between the expression vectors. The luciferase expression vector had the CAH1 SP positioned at the N-terminal portion of the protein and is not based in a fused protein, while our pJP28 vector was based on a fusion protein with a 2A self-cleaving peptide before the CAH1 SP. We could confirm that pJP34 SP interfered with the fusion protein cleavage, demonstrating that the amino acid sequences in different parts of the fused protein can interact and interfere with each other. It may be that the low fluorescence values are due to some intra sequence interactions inhibiting proper protein expression, in the context of our vector design. Nevertheless, these findings cannot be directly extrapolated to CAH1 SP, because we did not detect a fused form on the western blot.

Superior mCherry fluorescence is expected from the non-SP construct pAH04 [8], which may be associated with bottlenecks in the secretory pathway [15]. Notably, protein secretion is a multi-step process, with a strong quality control system that is dependent on the protein complex, which can be engineered to increase secretion [15]. In fact, overexpression of secretion-related proteins in other expression systems prompted an increase in secretion [17,46] and will be tested in microalgae. Yet, SP constructs pJP30 and pJP32 identified *in silico* displayed comparable mCherry production, with 70% mCherry fluorescence compared with the non-secreting pAH04 construct (Fig 2), and overexpression of secretion-related proteins might reduce the difference in production between transformants or even demonstrate higher production between secretion strategies. Another interesting result demonstrated by the non-secreting pAH04 control, was the possibility of cell lysis, which might affect the secretion of mCherry. Initially, the mCherry fluorescence results (Fig 3) showed that only pJP30 and pJP32 transformants secreted proteins at levels significantly higher than that of the non-secreting control; however, upon evaluation of the relative mCherry fluorescence signal in the supernatant, it is possible to conclude the efficient secretion of all pJP transformants, except for pJP34 (Fig 1). We assessed the lysis hypothesis due to suboptimal growth conditions, by growing top producers in an orbital agitate flask, resulting in a markedly lower mCherry fluorescence signal in the pAH04 supernatant (S2 Fig).

As noted, we compared the SPs in a 96-well plate format under apparently suboptimal conditions that might influence the detection of mCherry in the supernatant. Still, this strategy allows several transformants to be compared, and might reduce sampling bias, while allowing SP efficiency to be distinguished. Nevertheless, it is possible to reduce the suboptimal growth obtained in a 96-well plate format, and to exploit its high-throughput advantages, such as the use of high-speed (>800 rpm) orbital shakers designed for microplates [57]. Another alternative is the use of cytometers, commonly used in the comparison of DNA sequences influencing protein expression [10]. However, technically, it would be complicated to correlate the results obtained by cytometry with secretion efficiency, as the fluorescence signal would only correlate with the intracellular protein. However, the use of efficient tandem fluorescent timers [58] might allow the secretion efficiency to be correlated by comparing the fluorescence ratio of the paired fluorescent proteins. The fluorescent protein that matures the fastest will present a higher relative fluorescence in fast secreting cells, since it will fluoresce earlier. Considering both the fluorescence ratio and total fluorescence, it is possible to determine high-secreting cells. Both strategies might increase the sensitivity of the test, but would likely lead to the same conclusions.

In summary, different SPs vary in their efficiency to promote protein secretion in *C*. *reinhardtii*, as previously described in other systems [28], and these should be carefully selected for use in expression vectors. The SPs tested herein provide a portfolio of options that may be used to promote secretion in *C*. *reinhardtii*, and will support the development of more efficient secretion system in this microalga. In addition, further investigation of the potential SPs available in the published dataset (S1 Dataset) may result in the identification of more efficient SPs and should be pursued. Such increased secretion efficiency will contribute to the development of this expression system, rendering it an even more attractive system for protein production, and its industrial application.

## Materials and methods

### Assembly of transformation vectors

All short oligos (33–60 bp) for SLiCE reactions were synthetized by Valuegene (San Diego, CA, US). All restriction enzymes were purchased from New England Biolabs (Ipswich, MA, US). The pAH04 vector is a modification of the pBR9 vector [41], except that pAH04 contains three *rbcS2* introns incorporated into the vector in the natural order found in the *rbcS2* gene, as described in S5 Fig, to enhance expression [29]. pAH04 was constructed in pBlueScript II (pBSII). To generate pJP constructs, SLiCE reactions [59] were performed in the pAH04 vector by seamlessly incorporating each SP (Table 1) into the 3’ portion of the Ble-2A fusion peptide, maintaining the XhoI restriction site immediately after the SP sequence.

SPSPs were selected from four types of secreted protein described in the literature: binding protein 1 –BiP1 [35]; arylsulfatase 1 –ARS1 [8], carbonic anhydrase 1 –CAH1 [25], and ice-binding protein 1 –IBP1 [36], and six *in silico* identified sequences. SignalP4.0 was used to predict SP sequences from a *C*. *reinhardtii* protein sequence dataset [37], as described in S6 Fig. The assembly v4.0, dataset Chlre4_best_protein.fasta, from the DOE Joint Genome Institute databank was used [38]. The protein sequences dataset submitted to the SignalP4.0, generated a dataset of 8556 possible SPs. The *in silico* identified SPs were submitted to a multiple sequence alignment by CLUSTALW in MEGA7 [60] and used to generate a phylogenetic tree by neighbor-joining method [61]. The position of the previously described SPs selected (BiP1, ARS1 and CAH1) were checked. The 6 SPs were selected in different positions on the phylogenetic tree for diversity, except for pJP30 and pJP35, which were selected close to the ARS1 position, a previously tested SP in our expression construction design [8].

### SPSPCulture conditions and *C*. *reinhardtii* transformation

All experiments were performed in the *C*. *reinhardtii* cc1690 strain, which has an intact cell wall (Sager 21 gr; Chlamydomonas Stock Center, St. Paul, MN, USA). Transformation of cc1690 cells was achieved by electroporation, as previously described [8]. Briefly, cells were grown to mid-log phase density (3–6 × 10^6^ cells/mL) in a TAP medium [62] at 25°C under constant illumination of 50 μmol photons/m^2^s at 150 rpm on a rotary shaker. Cells were pelleted by centrifugation and resuspended to 3–6 × 10^8^ cells/mL in a TAP medium supplemented with 40 mM sucrose. Cells (250 μL) and 500 ng of double-digested (*Xba*I and *Kpn*I) vector plasmid were incubated for 5–10 min on ice in a 4-mm cuvette. GenePulser XCell^TM^ (BioRad, Hercules, CA) was used to electroporate the cell/vector mix, with an exponential electric pulse of 2000 V/cm, with a set capacitance of 25 mF and no shunt resistor. Electroporated cells were resuspended in 10 mL of TAP/40 mM sucrose medium for 18 h. After recovery, the cells were pelleted by centrifugation at 2000 g for 10 min, and resuspended in 600 μL of TAP medium. Equal numbers of cells were added to two TAP/agar plates supplemented with 5 and 10 μg/mL zeocin, respectively. We incubated the cells until colonies were observable. dx.doi.org/10.17504/protocols.io.kfkctkw

### mCherry fluorescence analysis

To assess the secretion efficiency of the transformants, 96 colonies from the selection plates were evaluated as described in S7 Fig. We picked transformed colonies and cultured in 500 μL of TAP medium for 7 d in deep-well plates (Corning Axygen®, No.: PDW500CS, Thermo Fisher Scientific Inc., Waltham, MA), covered with Breathe-Easy® (Sigma-Aldrich®). Cultivation was performed on a rotary shaker, set to 150 rpm, under constant illumination (50 μmol photons/m^2^s). Then, 100 μL of each sample was transferred to a clear bottom 96-well plate (Corning Costar, Tewksbury, MA, USA) and fluorescence was measured using an Infinite® M200 PRO plate reader (Tecan, Männedorf, Switzerland). Fluorescence was measured at excitation 575/9 nm and emission 608/20 nm. Supernatant samples were obtained by spinning deep-well plates at 3000 × *g* for 10 min and transferring 100 μL from each well to the clear bottom 96-well plate (Corning Costar, Tewksbury, MA, USA), followed by fluorescence measurement. No normalization was conducted for any mCherry fluorescence, but the chlorophyll contents were checked to infer successful cell growth. 10.17504/protocols.io.kfnctme

### Protein relative abundance

To estimate mCherry protein abundance, we separated proteins by SDS-PAGE, and stained using the colloidal Coomassie method [63]. Images of the gels were recorded and analyzed with Fiji, an ImageJ distribution software [64,65]. We performed densitometry analysis in all lanes, by selecting the entire lane and comparing band intensity. The area under the mCherry peak was calculated using ImageJ, and compared with the total area of all peaks inside the lane.

### Western blotting

mCherry proteins were identified in the supernatant and lysate samples using an anti-RFP antibody [41]. Transgenic *C*. *reinhardtii* cultures were inoculated at 1 × 10^5^ cells/mL and grown for 7 days. Culture supernatant was recovered, and cells were lysed by sonication in lysis buffer [50 mM Tris·HCl (pH 8.0), 0.1% Triton X-100]. Intracellular soluble proteins were obtained by high-speed centrifugation at 20,000 × *g*. To visualize, 30 μg of soluble proteins was loaded into each well. Each sample was separated by 12% sodium dodecyl sulfate polyacrylamide gel electrophoresis (SDS-PAGE), which was performed under reducing conditions and then transferred to a nitrocellulose membrane (Pall Corporation; NY, USA). After blocking with 5% milk, membranes were probed with rabbit anti-RFP (Rockland, Gilbertsville, PA, USA), washed with TBST (0.2 M Tris, 1.37 M NaCl, 0.1% Tween-20, pH 7.6), followed by goat anti-rabbit antibody conjugated to alkaline phosphatase (1:5000). 10.17504/protocols.io.kfpctmn

### Intracellular fluorescence localization

Transformed strains were grown in TAP medium to the late log phase on a rotary shaker. Live cells were plated on TAP/1% agarose pads prior to image acquisition. Life-cell imaging was performed with a confocal fluorescence microscope to observe mCherry in the secretion vacuoles. mCherry fluorescence compartmentalization was observed by a confocal Zeiss LSM 780-NLO microscope, using an argon laser at 543 nm to excite mCherry and a spectral detector set at approximately 610–650 nm. For chlorophyll, we used a laser at 405 nm for excitation, and the spectral detector was set to 680 nm. All pictures were taken using the same system configuration and analyzed by Fiji, an ImageJ distribution software [64,65]. Images of cells were acquired at 0.4-μm distances in each channel in the z-axis. Then, images were stacked using the Fiji software Z projects function, generating the final images. Raw czi files are deposit at Zenodo (DOI 10.5281/zenodo.600682). 10.17504/protocols.io.kfrctm6

### Statistical analysis

All constructs were transformed in the cc1690 strain, and 96 colonies of each transformation were collected and evaluated by fluorescence measurements once, resulting in 96 independent data points for each construct. The fluorescence results are expressed as boxplot of the positive colonies. Strains were classified as positive when the fluorescence measurement was superior to the average of 96 independent wild-type replicates, within three standard deviations. To compare the constructs, R Statistic version 3.3.3 was used to perform one-way ANOVA (with Tukey’s test), and to test statistical hypotheses, the significance level was set at 0.05. Graphs were generated in RStudio v1.0.136; data points and the code used are deposited at Zenodo (S2 Dataset). For the results of flask cultures, errors bars indicate the standard deviation of three technical replicates for each strain.

## Supporting information

S1 Fig Phylogenetic tree. The tree was inferred using the neighbor-joining method [61]. The optimal tree with the sum of branch length = 847.29541226 is shown. The tree is drawn to scale, with branch lengths in the same units as those of the evolutionary distances used to infer the phylogenetic tree. The evolutionary distances were computed using the Poisson correction method [66] and units are expressed as the number of amino acid substitutions per site. The analysis involved 8,429 amino acid sequences. All positions containing gaps and missing data were eliminated. There were a total of 10 positions in the final dataset. Evolutionary analyses were conducted in MEGA7 [60]. pJP22 (white)–construct with arylsulfatase 1 SP; pJP26 (white)–construct with binding protein 1 SP; pJP28 (white)–construct with carbonic anhydrase 1 SP; pJP29 (black)–construct with ice-binding protein 1 SP; pJP30-35 (orange)–construct with *in silico* identified SP.
(TIF)

S2 Fig Compared mCherry fluorescence in 7-day culture. mCherry fluorescence in the supernatant and the whole culture after 7 d cultivation. pAH04 –construct without SP; pJP22 –construct with arylsulfatase 1 SP; pJP26 –construct with binding protein 1 SP; pJP28 –construct with carbonic anhydrase 1 SP; pJP29 –construct with ice-binding protein 1 SP; pJP30-35 –construct with *in silico* identified SP.
(TIF)

S3 Fig mCherry relative abundance by SDS-Coomassie. A) Supernatant sample concentrated by ultrafiltration (10 kDa); B) Cell lysate samples; mChe: mCherry band; WT: cc1690 parental wild-type strain; pAH04 –construct without SP; pJP22 –construct with arylsulfatase 1 SP; pJP26 –construct with binding protein 1 SP; pJP28 –construct with carbonic anhydrase 1 SP; pJP29 –construct with ice-binding protein 1 SP; pJP30-35 –construct with *in silico* identified SP.
(TIF)

S4 Fig Pattern comparison of mCherry present in the cytoplasm and secretory pathway. Cytoplasmic pattern displayed by pAH04 –construct without SP; Secretory pathway pattern displayed by pJP26 –construct with binding protein 1 SP. White line was artificially draw on the expected cytoplasmic membrane position. Live cells were plated on agar pads and images were acquired 0.4-μm apart in each channel in the z-axis. Then, images were stacked using the Fiji software Z projects function, generating the final images. An argon laser at 543 nm was used to excite mCherry, and a spectral detector set at approximately 610–650 nm was used to detect emitted fluorescence. All images were processed identically. Scale bar = 5 μm.
(TIF)

S5 Fig Constructs used for the nuclear expression of mCherry in *Chlamydomonas reinhardtii*. Vector maps represent the constructs used in the study. All vectors are comprised of P_AR1_ promoter, sh-ble bleomycin resistance marker, 2A FMDV 2A self-cleaving peptide, the mCherry fluorescent protein coding sequence, rbcS2 terminator region, and introns in the order that they occur in the rbcS2 gene. pAH04 –non-secreting construct. pJP–secreting constructs with different SPs at the SP position.
(TIF)

S6 Fig *In silico* SP identification by SignalP 4.0 from *C*. *reinhardtii* protein sequence dataset. Dataset used: Chlre4_best_protein.fasta. SignalP 4.0 software identifies possible SPs in protein sequences. The datasets used and created are available in the S1 Dataset.
(TIF)

S7 Fig Construct evaluation workflow. Wild-type cc1690 was transformed by electroporation with double-digested constructs, and distributed in zeocin supplemented TAP/agar plates after recovery. Then, single colonies for each construct were picked and added to a well containing 500 μL of liquid TAP media and sealed with Breathe-Easy®. Cells were grown for 7 d in a rotary shaker under constant illumination. Aliquots of the whole culture and supernatant were collected and mCherry fluorescence was determined.
(TIF)

S1 Table Analysis of mCherry fluorescence variation on transformants for each construct. (DOCX)

S1 Dataset *Chlamydomonas reinhardtii* theoretical signal peptides identified by SignalP 4.0 in the dataset from "The Genome Portal of the Department of Energy Joint Genome Institute" (http://genome.jgi.doe.gov/). DOI 10.5281/zenodo.603927.
(RAR)

S2 Dataset Raw fluorescence measurements of *Chlamydomonas reinhardtii* strains expressing mCherry and R code used to analysis. DOI 10.5281/zenodo.604643.
(RAR)

S3 Dataset mCherry fluorescence correlation with western blot band intensity. DOI 10.5281/zenodo.1118977.
(RAR)

## References

[1] Borowitzka MA. High-value products from microalgae-their development and commercialisation. J Appl Phycol. 2013;25: 743–756. doi: 10.1007/s10811-013-9983-9

[2] Gouveia L, Oliveira AC. Microalgae as a raw material for biofuels production. J Ind Microbiol Biotechnol. 2009;36: 269–274. doi: 10.1007/s10295-008-0495-6 18982369 10.1007/s10295-008-0495-6

[3] Enzing C, Ploeg M, Barbosa M, Sijtsma L. Microalgae-based products for the food and feed sector: an outlook for Europe. JRC Scientific and Policy Reports. European Comission. 2014 doi: 10.2791/3339

[4] Tusé D, Tu T, McDonald KA. Manufacturing economics of plant-made biologics: Case studies in therapeutic and industrial enzymes. BioMed Res Int. 2014;2014 doi: 10.1155/2014/256135 24977145 10.1155/2014/256135 PMC4058100

[5] Klutz S, Holtmann L, Lobedann M, Schembecker G. Cost evaluation of antibody production processes in different operation modes. Chem Eng Sci. Elsevier; 2016;141: 63–74. doi: 10.1016/j.ces.2015.10.029

[6] Tran M, Van C, Barrera DJ, Pettersson PL, Peinado CD, Bui J, et al Production of unique immunotoxin cancer therapeutics in algal chloroplasts. PNAS. 2012;110: E15–E22. doi: 10.1073/pnas.1214638110 23236148 10.1073/pnas.1214638110 PMC3538218

[7] Scranton MA, Ostrand JT, Fields FJ, Mayfield SP. Chlamydomonas as a model for biofuels and bio-products production. Plant J. 2015;82: 523–531. doi: 10.1111/tpj.12780 25641390 10.1111/tpj.12780 PMC5531182

[8] Rasala BA, Lee PA, Shen Z, Briggs SP, Mendez M, Mayfield SP. Robust Expression and Secretion of Xylanase 1 in Chlamydomonas reinhardtii by fusion to a selection Gene and processing with the FMDV 2A peptide. PLoS One. 2012;7: e43349 doi: 10.1371/journal.pone.0043349 22937037 10.1371/journal.pone.0043349 PMC3427385

[9] Lauersen KJ, Kruse O, Mussgnug JH. Targeted expression of nuclear transgenes in Chlamydomonas reinhardtii with a versatile, modular vector toolkit. Appl Microbiol Biotechnol. 2015;99: 3491–3503. doi: 10.1007/s00253-014-6354-7 25586579 10.1007/s00253-014-6354-7

[10] Scranton MA, Ostrand JT, Georgianna DR, Lofgren SM, Li D, Ellis RC, et al Synthetic promoters capable of driving robust nuclear gene expression in the green alga Chlamydomonas reinhardtii. Algal Res. 2016;15: 135–142. doi: 10.1016/j.algal.2016.02.011

[11] Remacle C, Cardol P, Coosemans N, Gaisne M, Bonnefoy N. High-efficiency biolistic transformation of Chlamydomonas mitochondria can be used to insert mutations in complex I genes. Proc Natl Acad Sci U S A. 2006;103: 4771–6. doi: 10.1073/pnas.0509501103 16537419 10.1073/pnas.0509501103 PMC1450245

[12] Mathieu-Rivet E, Scholz M, Arias C, Dardelle F, Schulze S, Le Mauff F, et al Exploring the N-glycosylation pathway in Chlamydomonas reinhardtii unravels novel complex structures. Mol Cell Proteomics. 2013;12: 3160–83. doi: 10.1074/mcp.M113.028191 23912651 10.1074/mcp.M113.028191 PMC3820931

[13] Shin S-E, Lim J-M, Koh HG, Kim EK, Kang NK, Jeon S, et al CRISPR/Cas9-induced knockout and knock-in mutations in Chlamydomonas reinhardtii. Sci Rep. 2016;6: 27810 doi: 10.1038/srep27810 27291619 10.1038/srep27810 PMC4904240

[14] Diaz-Santos E, Vila M, Vigara J, León R. A new approach to express transgenes in microalgae and its use to increase the flocculation ability of Chlamydomonas reinhardtii. J Appl Phycol. 2016;28: 1611–1621. doi: 10.1007/s10811-015-0706-2

[15] Idiris A, Tohda H, Kumagai H, Takegawa K. Engineering of protein secretion in yeast: strategies and impact on protein production. Appl Microbiol Biotechnol. 2010;86: 403–17. doi: 10.1007/s00253-010-2447-0 20140428 10.1007/s00253-010-2447-0

[16] Ward OP. Production of recombinant proteins by filamentous fungi. Biotechnol Adv. 2012;30: 1119–1139. doi: 10.1016/j.biotechadv.2011.09.012 21968147 10.1016/j.biotechadv.2011.09.012

[17] Ahmad M, Hirz M, Pichler H, Schwab H. Protein expression in Pichia pastoris: Recent achievements and perspectives for heterologous protein production. Appl Microbiol Biotechnol. 2014;98: 5301–5317. doi: 10.1007/s00253-014-5732-5 24743983 10.1007/s00253-014-5732-5 PMC4047484

[18] Shukla AA, Thömmes J. Recent advances in large-scale production of monoclonal antibodies and related proteins. Trends Biotechnol. 2010;28: 253–261. doi: 10.1016/j.tibtech.2010.02.001 20304511 10.1016/j.tibtech.2010.02.001

[19] Tyo KEJ, Liu Z, Magnusson Y, Petranovic D, Nielsen J. Impact of protein uptake and degradation on recombinant protein secretion in yeast. ApplMicrobiol Biotechnol. 2014;98: 7149–7159. doi: 10.1007/s00253-014-5783-7 24816620 10.1007/s00253-014-5783-7

[20] Pollock J, Ho S V., Farid SS. Fed-batch and perfusion culture processes: Economic, environmental, and operational feasibility under uncertainty. Biotechnol Bioeng. 2013;110: 206–219. doi: 10.1002/bit.24608 22806692 10.1002/bit.24608

[21] Ramos-Martinez E, Fimognari L, Sakuragi Y. High yield secretion of recombinant proteins from the microalga Chlamydomonas reinhardtii. Plant Biotechnol J. 2017;15: 42–49. doi: 10.1111/pbi.12710 28207991 10.1111/pbi.12710 PMC5552477

[22] Hegde RS, Bernstein HD. The surprising complexity of signal sequences. Trends Biochem Sci. 2006;31: 563–71. doi: 10.1016/j.tibs.2006.08.004 16919958 10.1016/j.tibs.2006.08.004

[23] Kang Z, Zhang N, Zhang Y. Enhanced production of leech hyaluronidase by optimizing secretion and cultivation in Pichia pastoris. Appl Microbiol Biotechnol. 2016;100: 707–717. doi: 10.1007/s00253-015-7056-5 26476646 10.1007/s00253-015-7056-5

[24] Lin-Cereghino GP, Stark CM, Kim D, Chang J, Shaheen N, Poerwanto H, et al The effect of α-mating factor secretion signal mutations on recombinant protein expression in Pichia pastoris. Gene. 2013;519: 311–317. doi: 10.1016/j.gene.2013.01.062 23454485 10.1016/j.gene.2013.01.062 PMC3628533

[25] Lauersen KJ, Berger H, Mussgnug JH, Kruse O. Efficient recombinant protein production and secretion from nuclear transgenes in Chlamydomonas reinhardtii. J Biotechnol. 2013;167: 101–10. doi: 10.1016/j.jbiotec.2012.10.010 23099045 10.1016/j.jbiotec.2012.10.010

[26] Hamilton SR, Davidson RC, Sethuraman N, Nett JH, Jiang Y, Rios S, et al Humanization of yeast to produce complex terminally sialylated glycoproteins. Science. 2006;313: 1441–1443. doi: 10.1126/science.1130256 16960007 10.1126/science.1130256

[27] Knappskog S, Ravneberg H, Gjerdrum C, Tröße C, Stern B, Pryme IF. The level of synthesis and secretion of Gaussia princeps luciferase in transfected CHO cells is heavily dependent on the choice of signal peptide. J Biotechnol. 2007;128: 705–715. doi: 10.1016/j.jbiotec.2006.11.026 17316861 10.1016/j.jbiotec.2006.11.026

[28] Kober L, Zehe C, Bode J. Optimized signal peptides for the development of high expressing CHO cell lines. Biotechnol Bioeng. 2013;110: 1164–73. doi: 10.1002/bit.24776 23124363 10.1002/bit.24776

[29] Eichler-Stahlberg A, Weisheit W, Ruecker O, Heitzer M. Strategies to facilitate transgene expression in Chlamydomonas reinhardtii. Planta. 2009;229: 873–83. doi: 10.1007/s00425-008-0879-x 19127370 10.1007/s00425-008-0879-x

[30] Ruecker O, Zillner K, Groebner-Ferreira R, Heitzer M. Gaussia-luciferase as a sensitive reporter gene for monitoring promoter activity in the nucleus of the green alga Chlamydomonas reinhardtii. Mol Genet Genomics. 2008;280: 153–162. doi: 10.1007/s00438-008-0352-3 18516621 10.1007/s00438-008-0352-3

[31] Lauersen KJ, Vanderveer TL, Berger H, Kaluza I, Mussgnug JH, Walker VK, et al Ice recrystallization inhibition mediated by a nuclear-expressed and secreted recombinant ice-binding protein in the microalga Chlamydomonas reinhardtii. Appl Microbiol Biotechnol. 2013;97: 9763–72. doi: 10.1007/s00253-013-5226-x 24037309 10.1007/s00253-013-5226-x

[32] Lauersen KJ, Huber I, Wichmann J, Baier T, Leiter A, Gaukel V, et al Investigating the dynamics of recombinant protein secretion from a microalgal host. J Biotechnol. 2015;215: 62–71. doi: 10.1016/j.jbiotec.2015.05.001 25975624 10.1016/j.jbiotec.2015.05.001

[33] Ohresser M, Matagne RF, Loppes R. Expression of the arylsulphatase reporter gene under the control of the nit1 promoter in Chlamydomonas reinhardtii. Curr Genet. 1997;31: 264–271. doi: 10.1007/s002940050204 9065390 10.1007/s002940050204

[34] Baba M, Suzuki I, Shiraiwa Y. Proteomic analysis of high-CO(2)-inducible extracellular proteins in the unicellular green alga, Chlamydomonas reinhardtii. Plant Cell Physiol. 2011;52: 1302–14. doi: 10.1093/pcp/pcr078 21680606 10.1093/pcp/pcr078

[35] Rasala BA, Chao SS, Pier M, Barrera DJ, Mayfield SP. Enhanced genetic tools for engineering multigene traits into green algae. PLoS One. 2014;9 doi: 10.1371/journal.pone.0094028 24710110 10.1371/journal.pone.0094028 PMC3978050

[36] Raymond J A., Janech MG, Fritsen CH. Novel Ice-Binding Proteins From a Psychrophilic Antarctic Alga (Chlamydomonadaceae, Chlorophyceae). J Phycol. 2009;45: 130–136. doi: 10.1111/j.1529-8817.2008.00623.x 27033652 10.1111/j.1529-8817.2008.00623.x

[37] Petersen TN, Brunak S, von Heijne G, Nielsen H. SignalP 4.0: discriminating signal peptides from transmembrane regions. Nat Methods. 2011;8: 785–6. doi: 10.1038/nmeth.1701 21959131 10.1038/nmeth.1701

[38] Nordberg H, Cantor M, Dusheyko S, Hua S, Poliakov A, Shabalov I, et al The genome portal of the Department of Energy Joint Genome Institute: 2014 updates. Nucleic Acids Res. 2014;42: 26–31. doi: 10.1093/nar/gkt1069 24225321 10.1093/nar/gkt1069 PMC3965075

[39] Doron L, Segal N, Shapira M. Transgene Expression in Microalgae-From Tools to Applications. Front Plant Sci. 2016;7: 505 doi: 10.3389/fpls.2016.00505 27148328 10.3389/fpls.2016.00505 PMC4840263

[40] Fuhrmann M, Oertel W, Hegemann P. A synthetic gene coding for the green fluorescent protein (GFP) is a versatile reporter in Chlamydomonas reinhardtii. Plant J. 1999;19: 353–61. Available: http://www.ncbi.nlm.nih.gov/pubmed/10476082 10476082 10.1046/j.1365-313x.1999.00526.x

[41] Rasala B A, Barrera DJ, Ng J, Plucinak TM, Rosenberg JN, Weeks DP, et al Expanding the spectral palette of fluorescent proteins for the green microalga Chlamydomonas reinhardtii. Plant J. 2013;74: 545–56. doi: 10.1111/tpj.12165 23521393 10.1111/tpj.12165

[42] Vanier G, Hempel F, Chan P, Rodamer M, Vaudry D, Maier UG, et al Biochemical characterization of human anti-hepatitis b monoclonal antibody produced in the microalgae phaeodactylum tricornutum. PLoS One. 2015;10: 1–19. doi: 10.1371/journal.pone.0139282 26437211 10.1371/journal.pone.0139282 PMC4593558

[43] Hempel F, Maier UG. An engineered diatom acting like a plasma cell secreting human IgG antibodies with high efficiency. Microb Cell Fact. 2012;11: 126 doi: 10.1186/1475-2859-11-126 22970838 10.1186/1475-2859-11-126 PMC3503769

[44] Wolf J, Ross IL, Radzun KA, Jakob G, Stephens E, Hankamer B. High-throughput screen for high performance microalgae strain selection and integrated media design. Algal Res. Elsevier B.V.; 2015;11: 313–325. doi: 10.1016/j.algal.2015.07.005

[45] Baur A, Reski R, Gorr G. Enhanced recovery of a secreted recombinant human growth factor using stabilizing additives and by co-expression of human serum albumin in the moss Physcomitrella patens. Plant Biotechnol J. 2005;3: 331–340. doi: 10.1111/j.1467-7652.2005.00127.x 17129315 10.1111/j.1467-7652.2005.00127.x

[46] Harmsen MM, Bruyne MI, Raué HA, Maat J. Overexpression of binding protein and disruption of the PMR1 gene synergistically stimulate secretion of bovine prochymosin but not plant Thaumatin in yeast. Appl Microbiol Biotechnol. 1996;46: 365–370. doi: 10.1007/s002530050831 8987725 10.1007/BF00166231

[47] Klatt S, Konthur Z. Secretory signal peptide modification for optimized antibody-fragment expression-secretion in Leishmania tarentolae. Microb Cell Fact. 2012;11: 97 doi: 10.1186/1475-2859-11-97 22830363 10.1186/1475-2859-11-97 PMC3416730

[48] Mori A, Hara S, Sugahara T, Kojima T, Iwasaki Y, Kawarasaki Y, et al Signal peptide optimization tool for the secretion of recombinant protein from Saccharomyces cerevisiae. J Biosci Bioeng. 2015;120: 518–525. doi: 10.1016/j.jbiosc.2015.03.003 25912446 10.1016/j.jbiosc.2015.03.003

[49] Parekh RN, Wittrup KD. Expression level tuning for optimal heterologous protein secretion in Saccharomyces cerevisiae. Biotechnol Prog. 1997;13: 117–122. doi: 10.1021/bp970009d 9104035 10.1021/bp970009d

[50] Lodi T, Neglia B, Donnini C. Secretion of Human Serum Albumin by Kluyveromyces lactis Overexpressing KlPDI1 and KlERO1. Appl Env Microbiol. 2005;71: 4359–4363. doi: 10.1128/AEM.71.8.4359 16085825 10.1128/AEM.71.8.4359-4363.2005 PMC1183311

[51] Li P, Anumanthan A, Gao X-G, Ilangovan K, Suzara V V., Düzgüneş N, et al Expression of Recombinant Proteins in Pichia pastoris. Appl Biochem Biotechnol. 2007;142: 105–124. doi: 10.1007/s12010-007-0003-x 18025573 10.1007/s12010-007-0003-x

[52] Fuhrmann M, Hausherr A, Ferbitz L, Schödl T, Heitzer M, Hegemann P. Monitoring dynamic expression of nuclear genes in Chlamydomonas reinhardtii by using a synthetic luciferase reporter gene. Plant Mol Biol. 2004;55: 869–881. doi: 10.1007/s11103-004-2150-6 15604722 10.1007/s11103-004-2150-6

[53] Schroda M, Blöcker D, Beck CF. The HSP70A promoter as a tool for the improved expression of transgenes in Chlamydomonas. Plant J. 2000;21: 121–31. Available: http://www.ncbi.nlm.nih.gov/pubmed/10743653 10743653 10.1046/j.1365-313x.2000.00652.x

[54] Merchant SS, Prochnik SE, Vallon O, Harris EH, Karpowicz J, Witman GB, et al The Chlamydomonas Genome Reveals the Evolution of Key Animal and Plant Functions. Science. 2007;318: 245–250. doi: 10.1126/science.1143609 17932292 10.1126/science.1143609 PMC2875087

[55] Kong F, Yamasaki T, Kurniasih SD, Hou L, Li X, Ivanova N, et al Robust expression of heterologous genes by selection marker fusion system in improved Chlamydomonas strains. J Biosci Bioeng. 2015;120: 239–245. doi: 10.1016/j.jbiosc.2015.01.005 25660568 10.1016/j.jbiosc.2015.01.005

[56] Van K, Spalding MH. Periplasmic Carbonic Anhydrase Structural Gene (Cah1) Mutant in Chlamydomonas reinhardtii 1. Plant Physiol. 1999;120: 757–764. doi: 10.1104/pp.120.3.757 10398710 10.1104/pp.120.3.757 PMC59313

[57] Radzun KA, Wolf J, Jakob G, Zhang E, Stephens E, Ross I, et al Automated nutrient screening system enables high-throughput optimisation of microalgae production conditions. Biotechnol Biofuels. 2015;8: 65 doi: 10.1186/s13068-015-0238-7 25984234 10.1186/s13068-015-0238-7 PMC4432509

[58] Khmelinskii A, Keller PJ, Bartosik A, Meurer M, Barry JD, Mardin BR, et al Tandem fluorescent protein timers for in vivo analysis of protein dynamics. Nat Biotechnol. 2012;30: 708–14. doi: 10.1038/nbt.2281 22729030 10.1038/nbt.2281

[59] Zhang Y, Werling U, Edelmann W. SLiCE: a novel bacterial cell extract-based DNA cloning method. Nucleic Acids Res. 2012;40: e55 doi: 10.1093/nar/gkr1288 22241772 10.1093/nar/gkr1288 PMC3333860

[60] Kumar S, Stecher G, Tamura K. MEGA7: Molecular Evolutionary Genetics Analysis Version 7.0 for Bigger Datasets. Mol Biol Evol. 2016;33: 1870–1874. doi: 10.1093/molbev/msw054 27004904 10.1093/molbev/msw054 PMC8210823

[61] Saitou N, Nei M. The neighbour-joining method: a new method for reconstructing phylogenetic trees. Mol Biol Evo. 1987;4: 406–425. citeulike-article-id:93683. 10.1093/oxfordjournals.molbev.a040454 3447015

[62] Gorman DS, Levine RP. Cytochrome F and plastocyanin: their sequence in the photosynthetic electron transport chain of chlamydomonas reinhardtii. PNAS. 1965;54: 1665–1669. 4379719 10.1073/pnas.54.6.1665 PMC300531

[63] Dyballa N, Metzger S. Fast and sensitive colloidal coomassie G-250 staining for proteins in polyacrylamide gels. JoVE. 2009; 2–5. doi: 10.3791/1431 19684561 10.3791/1431 PMC3149902

[64] Abràmoff MD, Hospitals I, Magalhães PJ, Abràmoff M. Image Processing with ImageJ. Biophotonics Int. 2004;11: 36–42.

[65] Schindelin J, Arganda-Carreras I, Frise E, Kaynig V, Longair M, Pietzsch T, et al Fiji: an open-source platform for biological-image analysis. Nat Methods. 2012;9: 676–682. doi: 10.1038/nmeth.2019 22743772 10.1038/nmeth.2019 PMC3855844

[66] Zuckerkandl E, Pauling L. Evolutionary divergence and convergence in proteins. Evol Genes Proteins. 1965; 97–166. doi: 10.1209/epl/i1998-00224-x

