# Supplementary figures and images for: Comparison of secretory signal peptides for heterologous protein expression in microalgae: Expanding the secretion portfolio for Chlamydomonas reinhardtii
Source: PLoS One. 2018 Feb 6;13(2):e0192433. doi: 10.1371/journal.pone.0192433 (PMC5800701; doi:10.1371/journal.pone.0192433)

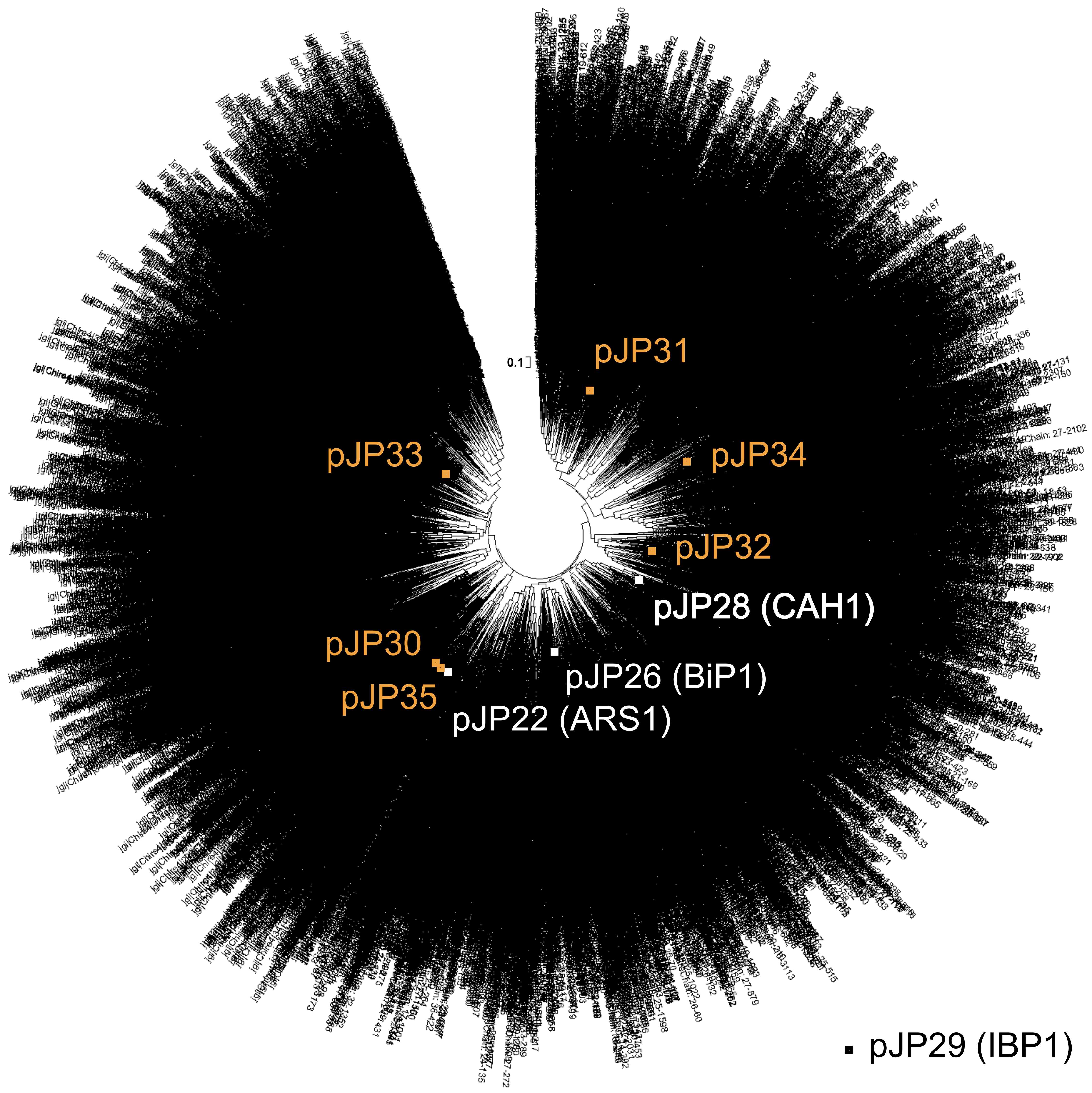

Supplement: S1 Fig — The tree was inferred using the neighbor-joining method [61]. The optimal tree with the sum of branch length = 847.29541226 is shown. The tree is drawn to scale, with branch lengths in the same units as those of the evolutionary distances used to infer the phylogenetic tree. The evolutionary distances were computed using the Poisson correction method [66] and units are expressed as the number of amino acid substitutions per site. The analysis involved 8,429 amino acid sequences. All positions containing gaps and missing data were eliminated. There were a total of 10 positions in the final dataset. Evolutionary analyses were conducted in MEGA7 [60]. pJP22 (white)–construct with arylsulfatase 1 SP; pJP26 (white)–construct with binding protein 1 SP; pJP28 (white)–construct with carbonic anhydrase 1 SP; pJP29 (black)–construct with ice-binding protein 1 SP; pJP30-35 (orange)–construct with in silico identified SP. (TIF) [file pone.0192433.s001.tif]

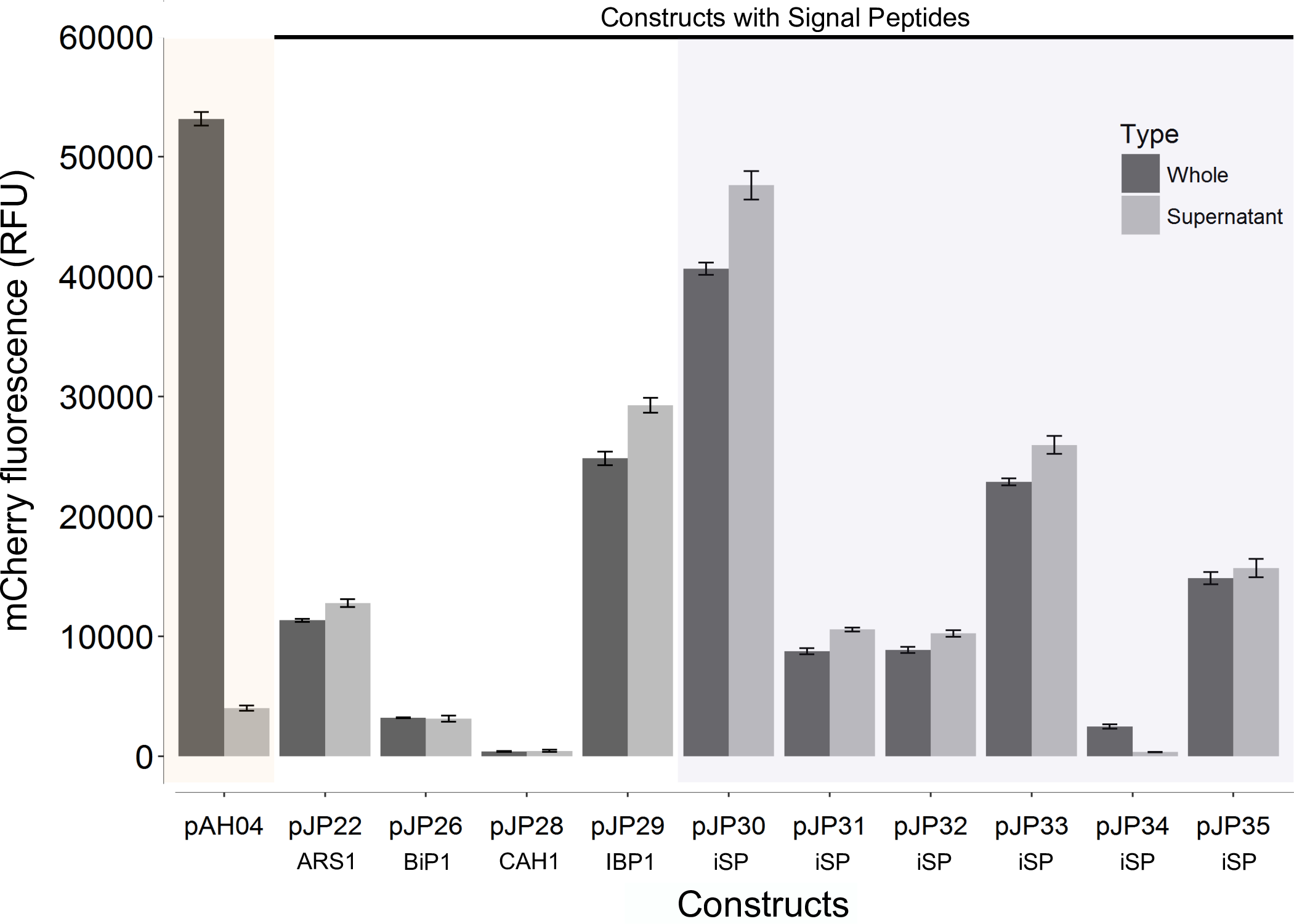

Supplement: S2 Fig — mCherry fluorescence in the supernatant and the whole culture after 7 d cultivation. pAH04 –construct without SP; pJP22 –construct with arylsulfatase 1 SP; pJP26 –construct with binding protein 1 SP; pJP28 –construct with carbonic anhydrase 1 SP; pJP29 –construct with ice-binding protein 1 SP; pJP30-35 –construct with in silico identified SP. (TIF) [file pone.0192433.s002.tif]

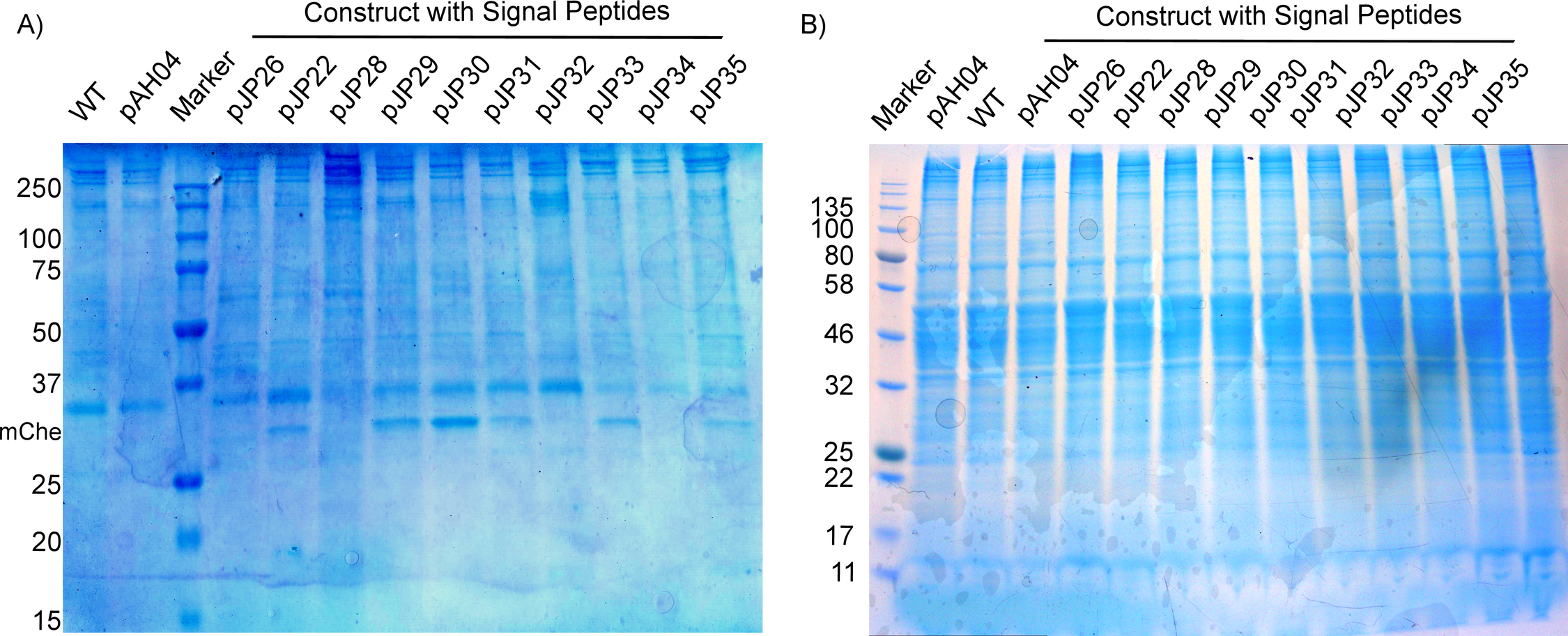

Supplement: S3 Fig — A) Supernatant sample concentrated by ultrafiltration (10 kDa); B) Cell lysate samples; mChe: mCherry band; WT: cc1690 parental wild-type strain; pAH04 –construct without SP; pJP22 –construct with arylsulfatase 1 SP; pJP26 –construct with binding protein 1 SP; pJP28 –construct with carbonic anhydrase 1 SP; pJP29 –construct with ice-binding protein 1 SP; pJP30-35 –construct with in silico identified SP. (TIF) [file pone.0192433.s003.tif]

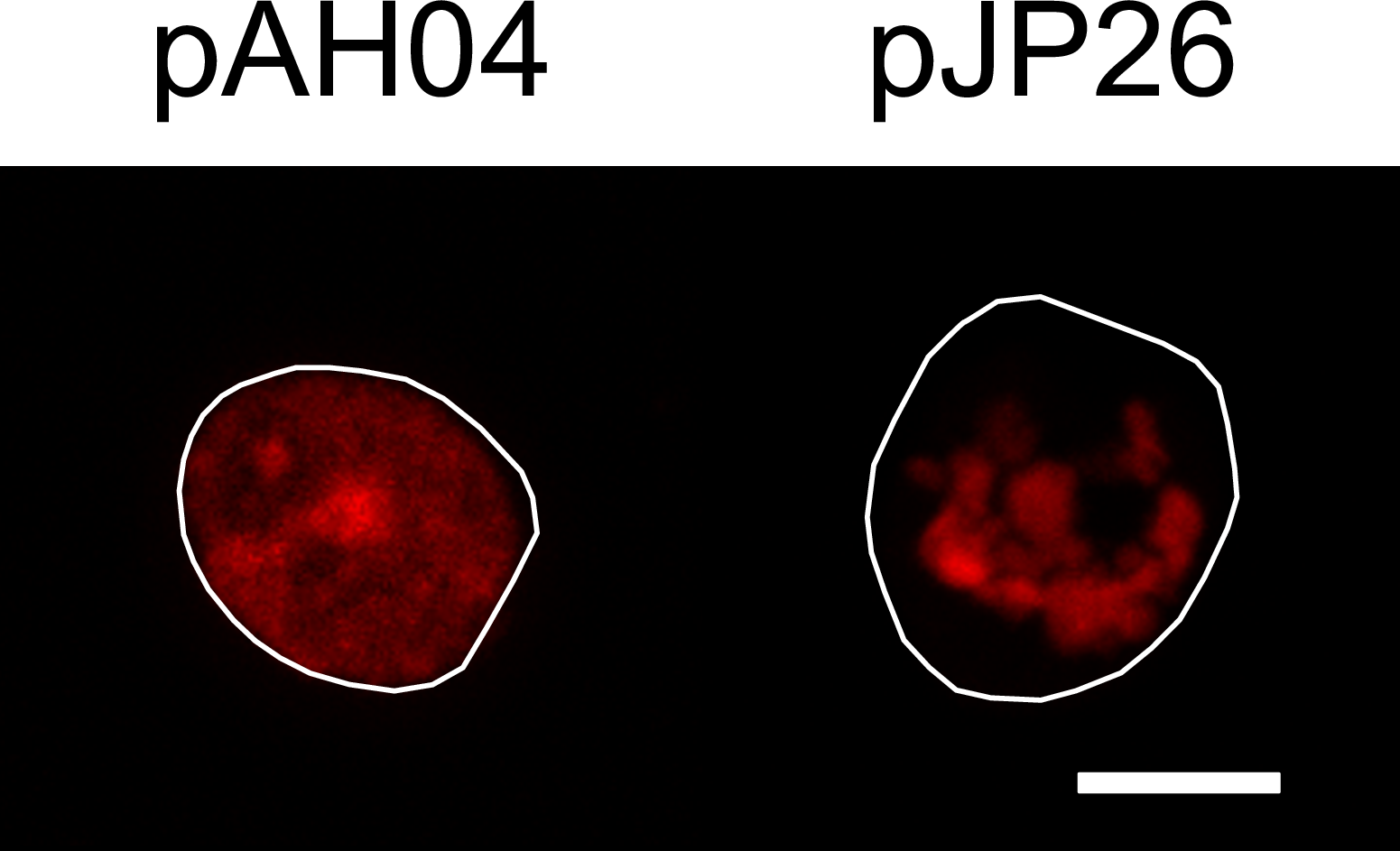

Supplement: S4 Fig — Cytoplasmic pattern displayed by pAH04 –construct without SP; Secretory pathway pattern displayed by pJP26 –construct with binding protein 1 SP. White line was artificially draw on the expected cytoplasmic membrane position. Live cells were plated on agar pads and images were acquired 0.4-μm apart in each channel in the z-axis. Then, images were stacked using the Fiji software Z projects function, generating the final images. An argon laser at 543 nm was used to excite mCherry, and a spectral detector set at approximately 610–650 nm was used to detect emitted fluorescence. All images were processed identically. Scale bar = 5 μm. (TIF) [file pone.0192433.s004.tif]

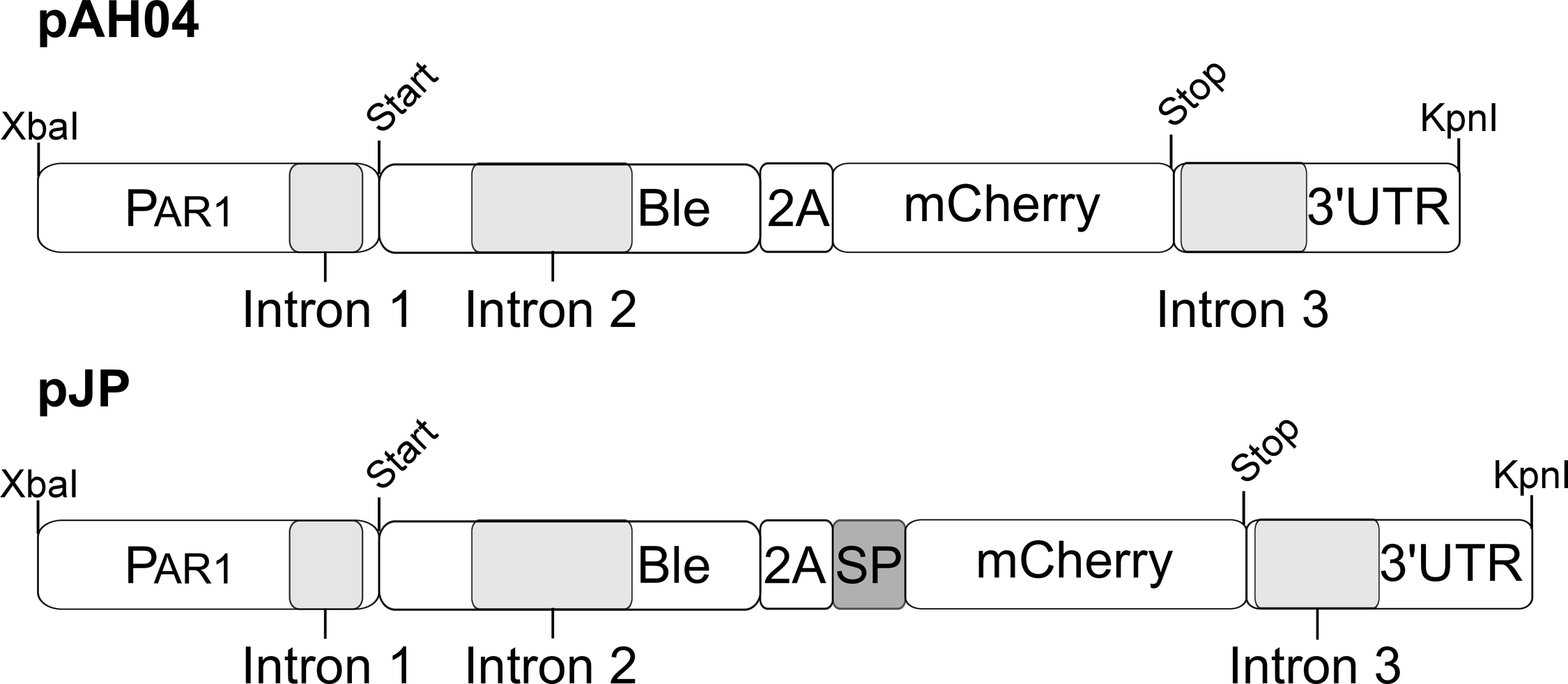

Supplement: S5 Fig — Vector maps represent the constructs used in the study. All vectors are comprised of PAR1 promoter, sh-ble bleomycin resistance marker, 2A FMDV 2A self-cleaving peptide, the mCherry fluorescent protein coding sequence, rbcS2 terminator region, and introns in the order that they occur in the rbcS2 gene. pAH04 –non-secreting construct. pJP–secreting constructs with different SPs at the SP position. (TIF) [file pone.0192433.s005.tif]

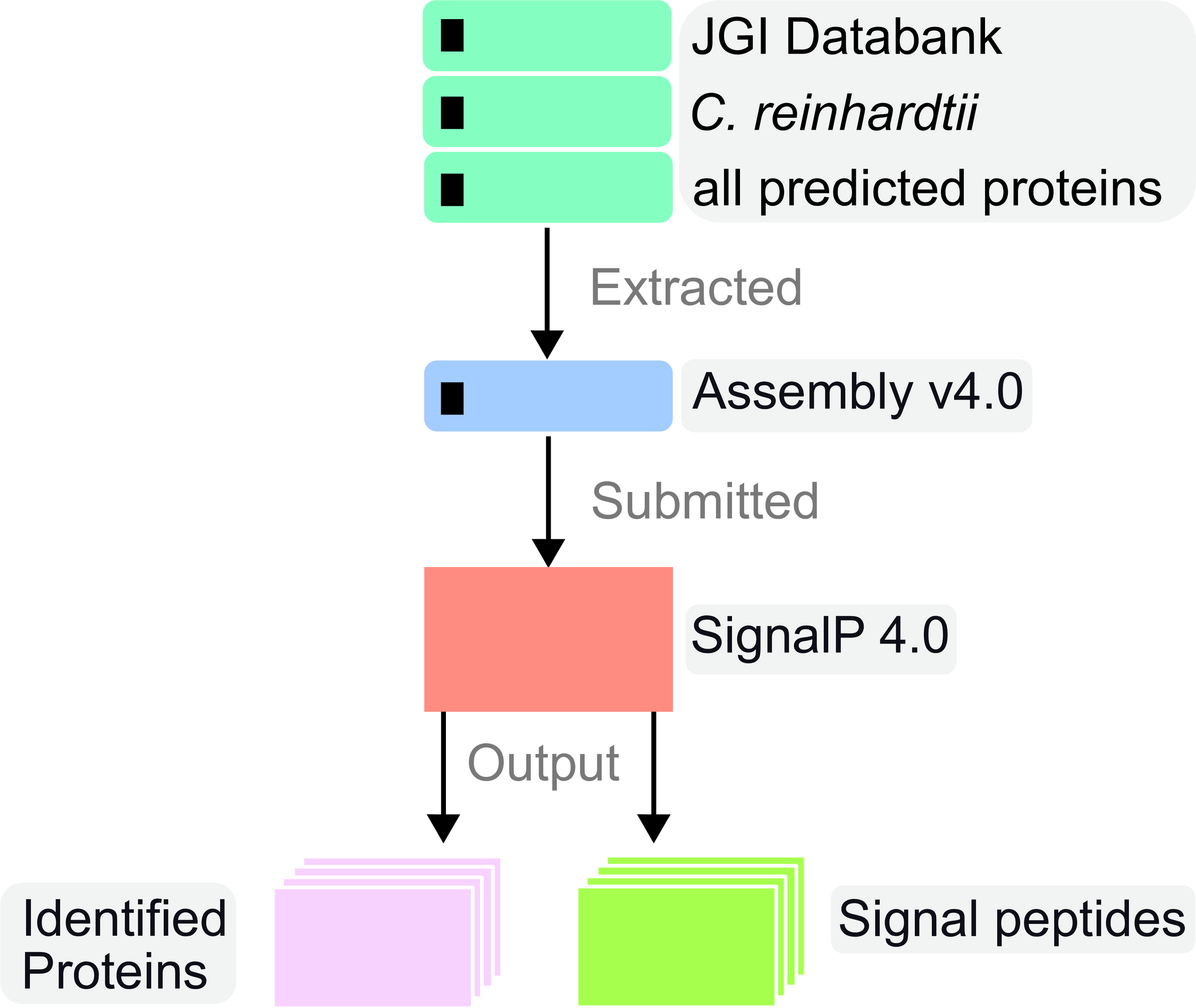

Supplement: S6 Fig — Dataset used: Chlre4_best_protein.fasta. SignalP 4.0 software identifies possible SPs in protein sequences. The datasets used and created are available in the S1 Dataset. (TIF) [file pone.0192433.s006.tif]

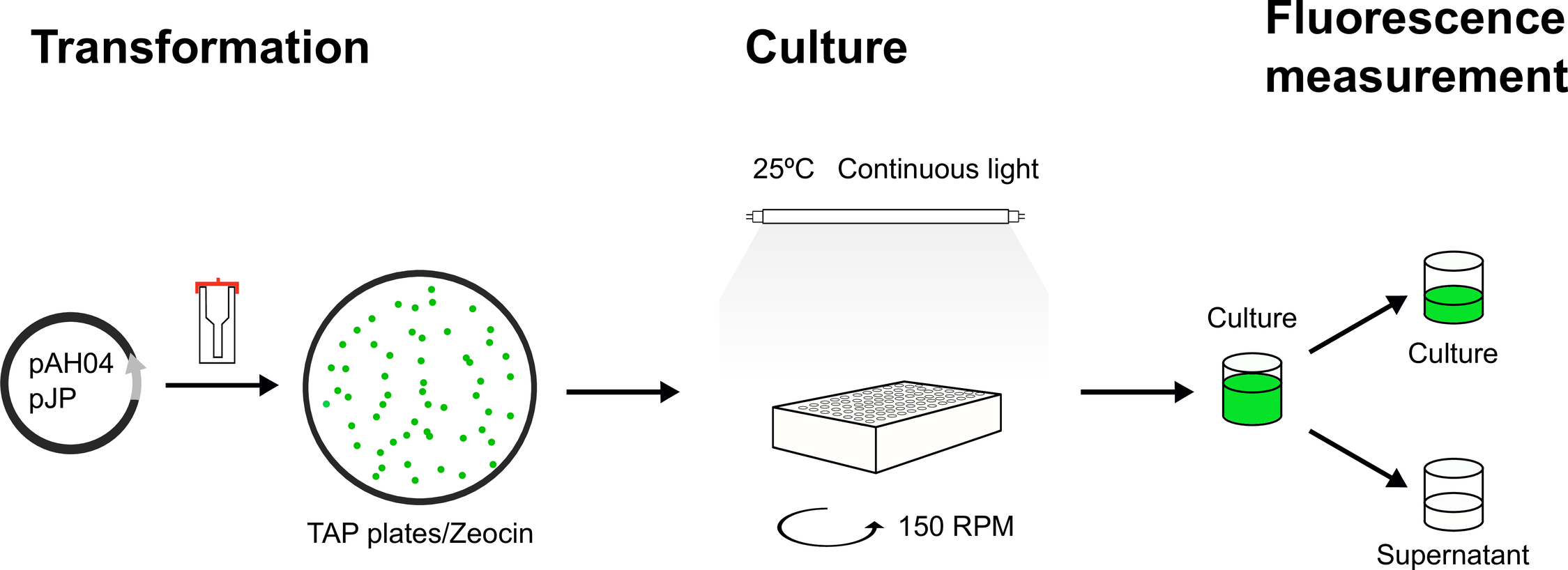

Supplement: S7 Fig — Wild-type cc1690 was transformed by electroporation with double-digested constructs, and distributed in zeocin supplemented TAP/agar plates after recovery. Then, single colonies for each construct were picked and added to a well containing 500 μL of liquid TAP media and sealed with Breathe-Easy®. Cells were grown for 7 d in a rotary shaker under constant illumination. Aliquots of the whole culture and supernatant were collected and mCherry fluorescence was determined. (TIF) [file pone.0192433.s007.tif]
