# Supplementary material for: Comparison of secretory signal peptides for heterologous protein expression in microalgae: Expanding the secretion portfolio for Chlamydomonas reinhardtii
Source: PLoS One. 2018 Feb 6;13(2):e0192433. doi: 10.1371/journal.pone.0192433 (PMC5800701; doi:10.1371/journal.pone.0192433)
Supplement: S1 Table — (DOCX) [file pone.0192433.s008.docx]

S1 Table: Analysis of mCherry fluorescence variation on transformants for each construct.

| Construct | Average RFU | STD (RFU) | RSD (%) |
| --- | --- | --- | --- |
| pAH04 | 18881 | 14418 | 76.4 |
| pJP26 | 8404 | 4790 | 57.0 |
| pJP22 | 11821 | 5514 | 46.6 |
| pJP28 | 1695 | 873 | 51.6 |
| pJP29 | 11304 | 11612 | 102.7 |
| pJP30 | 13718 | 8534 | 62.2 |
| pJP31 | 7912 | 4713 | 59.6 |
| pJP32 | 13908 | 5933 | 42.7 |
| pJP33 | 10404 | 5562 | 53.5 |
| pJP34 | 2407 | 1338 | 55.6 |
| pJP35 | 8739 | 4943 | 56.6 |

Note: RFU – Relative Fluorescence Unit; STD – Standard Deviation; RSD – Relative Standard Deviation.
